# Novel dynamics of human mucociliary differentiation revealed by single-cell RNA sequencing of nasal epithelial cultures

**DOI:** 10.1242/dev.177428

**Published:** 2019-10-15

**Authors:** Sandra Ruiz García, Marie Deprez, Kevin Lebrigand, Amélie Cavard, Agnès Paquet, Marie-Jeanne Arguel, Virginie Magnone, Marin Truchi, Ignacio Caballero, Sylvie Leroy, Charles-Hugo Marquette, Brice Marcet, Pascal Barbry, Laure-Emmanuelle Zaragosi

**Affiliations:** 1 Université Côte d'Azur, CNRS, IPMC, Sophia-Antipolis 06560, France; 2 ISP, INRA, Université Tours, Nouzilly 37380, France; 3 Université Côte d'Azur, CHU de Nice, Pulmonology Department, Nice 06000, France

**Keywords:** Airway epithelium, Single-cell RNA-seq, Differentiation, Multiciliated cells, Club cells, Goblet cells, Basal cells, Deuterosome, Keratins, Pathways

## Abstract

The upper airway epithelium, which is mainly composed of multiciliated, goblet, club and basal cells, ensures proper mucociliary function and can regenerate in response to assaults. In chronic airway diseases, defective repair leads to tissue remodeling. Delineating key drivers of differentiation dynamics can help understand how normal or pathological regeneration occurs. Using single-cell transcriptomics and lineage inference, we have unraveled trajectories from basal to luminal cells, providing novel markers for specific populations. We report that: (1) a precursor subgroup of multiciliated cells, which we have entitled deuterosomal cells, is defined by specific markers, such as DEUP1, FOXN4, YPEL1, HES6 and CDC20B; (2) goblet cells can be precursors of multiciliated cells, thus explaining the presence of hybrid cells that co-express markers of goblet and multiciliated cells; and (3) a repertoire of molecules involved in the regeneration process, such as keratins or components of the Notch, Wnt or BMP/TGFβ pathways, can be identified. Confirmation of our results on fresh human and pig airway samples, and on mouse tracheal cells, extend and confirm our conclusions regarding the molecular and cellular choreography at work during mucociliary epithelial differentiation.

## Abstract (precis)

**Highlighted Article:** Single-cell RNAseq data in fresh human airway epithelial tissues and air liquid cultures identifies novel cell populations and offers new insights into the molecular mechanisms occurring during mucociliary epithelium regeneration.

## INTRODUCTION

The airway epithelium makes an efficient line of defense against inhaled substances. It is mainly composed of multiciliated cells (MCCs), goblet cells (GCs), club cells (CCs) and basal cells (BCs) (Gras et al., 2013; Kotton and Morrisey, 2014). Decreased numbers of MCCs and increased number of GCs hallmark many chronic respiratory diseases, during which frequent injuries, repair defects, tissue remodeling and altered mucociliary clearance occur (Cohn, 2006; Curran and Cohn, 2010; Merigo et al., 2002). Characteristics contributing to efficient airway regeneration after injuries have been extensively investigated in mouse, establishing mouse BCs as the main airway stem cells, with self-renewal capacities and the ability to differentiate into MCCs, CCs and GCs (Cole et al., 2010; Kotton and Morrisey, 2014; Rock et al., 2009). BCs are abundant in upper mouse airways but absent from lower airways (Hogan et al., 2014). Human BCs populate the whole airways, and their abundance also decreases in smaller airways (Boers et al., 1998). A direct differentiation of BCs into MCCs has been reported after injury (Pardo-Saganta et al., 2015a), but the current consensus is that BCs can differentiate first into CCs (Watson et al., 2015), i.e. club/secretory or Clara cells. CCs are widespread in the whole mouse airways. They are less abundant in human, being nearly absent from upper airways but enriched in terminal and respiratory bronchioles (Boers et al., 1999). CCs are luminally located, show a characteristic columnar shape and contribute to xenobiotic metabolism through the production of anti-microbial and anti-inflammatory peptides (Wang et al., 2003; Jones et al., 1983), such as the secretoglobin SCGB1A1. CCs can give rise to MCCs, as detected by the expression of transcription factor FOXJ1 (Rawlins et al., 2009; Watson et al., 2015) and to GCs, as detected by the expression of mucin MUC5AC (Chen et al., 2009; Kotton and Morrisey, 2014).

Distinct molecular mechanisms regulate cell fate decisions in airway epithelium lineages. Notch signaling plays a pivotal role during commitment of BCs: activation leads to CC/GC lineages, while inhibition leads to MCC lineages (Morimoto et al., 2010; Pardo-Saganta et al., 2015b; Rock et al., 2011; Tsao et al., 2009). We have shown that Notch pathway inhibition by the *miR-34/449* families of microRNAs is required for MCC differentiation (Marcet et al., 2011a,b; Mercey et al., 2017). *In vivo* lineage-tracing studies have some limitations: observations in animal models do not necessarily transfer to human; use of drastic forms of injuries may not completely reveal physiological tissue turnover; and strategies of specific genetic cell labeling (usually *Krt5* for BCs and *Scgb1a1* for CCs) are not necessarily comprehensive and do not necessarily provide a full picture of the airway epithelial cell hierarchies. In human, in which lineage tracing is impossible, cell lineage hierarchies in homeostatic bronchi have been indirectly inferred by assessing somatic mitochondrial mutations (Teixeira et al., 2013); however, *in vitro* approaches are still necessary to study cell lineage during epithelial regeneration.

Single-cell RNA-sequencing has emerged as a powerful approach to measure cell lineage hierarchies (Fletcher et al., 2017; Karamitros et al., 2018; Pal et al., 2017), by capturing cells at different levels of differentiation (Plass et al., 2018). After a first study that delineated lineage hierarchies of mouse alveolar cells (Treutlein et al., 2014), several atlases of the airways have recently been released in mouse (Montoro et al., 2018) and human (Ordovas-Montanes et al., 2018; Plasschaert et al., 2018; Vieira Braga et al., 2019), providing a first panorama of human airway cell diversity and lineages that we are extending here, after analyzing single-cell RNA-seq data in fresh human airway epithelial tissues and throughout an experiment in 3D *in vitro* regeneration of human airway epithelium. The resulting cell trajectory roadmap of human airways identifies novel cell populations and offers new insights into molecular mechanisms taking place during the mucociliary epithelium regeneration.

## RESULTS

### Reconstruction of cell lineage in regenerating airway epithelium by single-cell RNA-seq

**Fig. 1.**
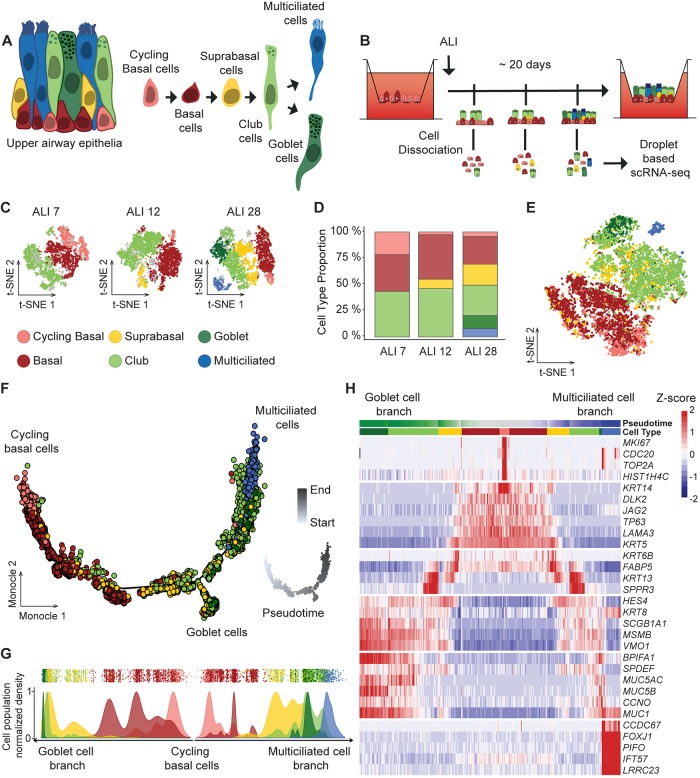
**Characterization of multiciliated and goblet cell lineages during airway epithelium regeneration using single-cell RNA-Seq.** (A) Model of upper airway epithelium, based on six major types of epithelial cells, with consensus lineage hierarchy. (B) scRNA-seq experimental design. Regenerating airway epithelia were dissociated on successive days (7, 12 and 28) after a transition to an air-liquid interface (ALI). (C) t-SNE plots of the scRNA-seq expression data highlighting the main cell types observed at ALI 7 (3426 cells), ALI 12 (2785 cells) and ALI 28 (3615 cells) (gray, unassigned cells). (D) Relative abundance of the six main cell types at each time point. (E) Aggregate t-SNE plot of gene expression in 9826 cells. (F) Inference of goblet and multiciliated cell lineages by Monocle 2, based on an aggregate of the entire experiment. Color code is the same as in C. Inset shows pseudotime picturing using a white-to-gray gradient along the differentiation trajectory. (G) Distribution of the six main cell types in the pseudotime along the two branches of the trajectory from F (bottom, goblet cell branch; top right, multiciliated cell branch). (H) Heatmap representing the smoothened temporal expression pattern of a representative list of cell type-specific markers, with branch representations as in G. Cells were ordered by branch, then cluster emergence, then pseudotime.

We have analyzed single-cell transcriptomes at successive stages during *in vitro* 3D differentiation of human airway epithelial cells (HAECs) (Fig. 1A,B). This *in vitro* model faithfully recapitulated cell population compositions found in native airway tissues, as shown by a comparison between single-cell (sc) RNA-seq of epithelial cells dissociated from nasal brushing samples or from fresh nasal turbinates and scRNA-seq of HAECs at a late time point of *in vitro* air-liquid interface differentiation (3D cells) (Fig. S1). Most of our results were obtained with HAECs that were differentiated in Pneumacult media (StemCell Technologies), which allows the production of multiciliated cells and goblet cells. Additional experiments were also performed with HAECs differentiated in BEGM (Lonza), which rather favors the production of multiciliated cells. Cell identity was inferred from the expression of specific marker genes, such as *KRT5* and *TP63* for basal cells (BCs), *SCGB1A1* for club cells (CCs), *MUC5AC* for goblet cells (GCs), and *FOXJ1* for multiciliated cells (MCCs). These cell types were robustly found in all samples at various proportions (Fig. S1A-C). We also confirmed that cell type proportions inferred from scRNA-seq were correlated with cell type proportions inferred from protein measurements by performing immunostaining of selected population markers (Fig. S1D,E). Cell dissociation did not produce a major impact on gene expression with the exception of *FOS* and *FOSB* (Fig. S2). Molecular function enrichment with Ingenuity Pathway Analysis (Qiagen) showed that ‘cell death and survival’ and ‘cellular growth and proliferation’ were the only molecular functions that were regulated with *P*<0.001 (Fig. S2C).

Single-cell transcriptomes of HAECs differentiated in Pneumacult medium were analyzed at three time points [after transition to an air-liquid interface (ALI) 7, ALI 12 and ALI 28] (Fig. 1B), which are representative of the proliferation, polarization and specification steps of regeneration (Chevalier et al., 2015). This experiment was complemented by six additional time points of HAECs differentiated in BEGM medium (ALI 2, ALI 4, ALI 7, ALI 12, ALI 17 and ALI 22). In the first approach, each time point was analyzed independently. We carried out 10 random selections of cells, corresponding to subgroups containing 90% of the initial number of cells. The resulting gene expression submatrices were then iteratively clustered (10 times with varying parameters), and a census was applied to define the most robust cell types. We then studied the variations of these populations during the entire time course. Cells clustered in six main populations in Pneumacult: (1) cycling (*MKI67*+) BCs; (2) non-cycling (*MKI67*−) BCs (*KRT5*+/*TP63*+); (3) supraBCs (*KRT5*+/*TP63*−/*KRT13*+/*KRT4*+); (4) CCs (*SCGB1A1*+); (5) GCs (*MUC5AC*+); and (6) MCCs (*FOXJ1*+) (Fig. 1C; Table S1). Cell population proportions evolved during the time course, with a global reduction in BCs and CCs, an initial detection of supraBCs at ALI 7, followed by an increase of the proportion of this cell population at ALI 28, and an initial detection of GCs and MCCs at ALI 28 (Fig. 1D). In BEGM, cells clustered in seven cell populations (Fig. S3A,B and Table S2). We did not detect CCs and GCs using this culture condition, but found instead a cell population that we termed ‘Club-like cells’, given their high gene expression similarity with CCs, except for *SCGB1A1*, which was not detected (Fig. S4). Additional cell types were found in these samples: *KRT5*− supraBCs (*TP63*−/*KRT13*+/*KRT4*+) and two cell populations that we termed as ‘undefined intermediates 1’ and ‘undefined intermediates 2’ because their gene expression profiles did not allow unambiguous classification. Inter-donor variability was assessed by analyzing ALI cultures from independent donors in both BEGM and Pneumacult media. Very similar cell population distributions were found across donors and differences between the two cell culture media were maintained in all samples (Fig. S5). An aggregated t-SNE graph for all cells at all time points for each medium condition was plotted (Pneumacult, Fig. 1E; BEGM, Fig. S3C). Cell trajectories and transitions from one cell population to another were deduced from a trajectory inference analysis using Monocle 2, followed by differential expression analysis between consecutive cell states in pseudotime using Seurat. Fig. S6 shows the position of all cells within pseudotime and trajectories color-coded according to their experimental time point of origin. In BEGM, a unique cell trajectory was found (Fig. S3D), starting with cycling and non-cycling BCs at its beginning, followed by *KRT5*+ and then *KRT5*− supraBCs cells, with MCCs at its end. Despite the absence of *SCGB1A1* expression in secretory-like cells (*SCGB1A1*−/*BPIFA1*+/*KRT8*+), these cells were ordered in the pseudotime before MCCs, as expected for canonical CCs (Fig. S3D-F). A more complex trajectory was observed with Pneumacult, in which Monocle 2 detected a bifurcation into two distinct branches after the SC stage: a larger branch leading to *FOXJ1*+ MCCs, and a smaller one leading to *MUC5AC*+ GCs (Fig. 1F,G). A closer examination of pseudotime ordering and differential gene expression (Fig. 1H) revealed that some *MUC5AC*+ cells were found on the MCC branch, after the GC bifurcation and that some *FOXJ1*+ cells retained expression of *MUC5AC*. Altogether, our findings confirm CCs as precursors of both MCCs and GCs. They also suggest that GCs can also act as MCC precursors in airway epithelial regeneration.

### Goblet cells can be differentiation intermediates for multiciliated cells

**Fig. 2.**
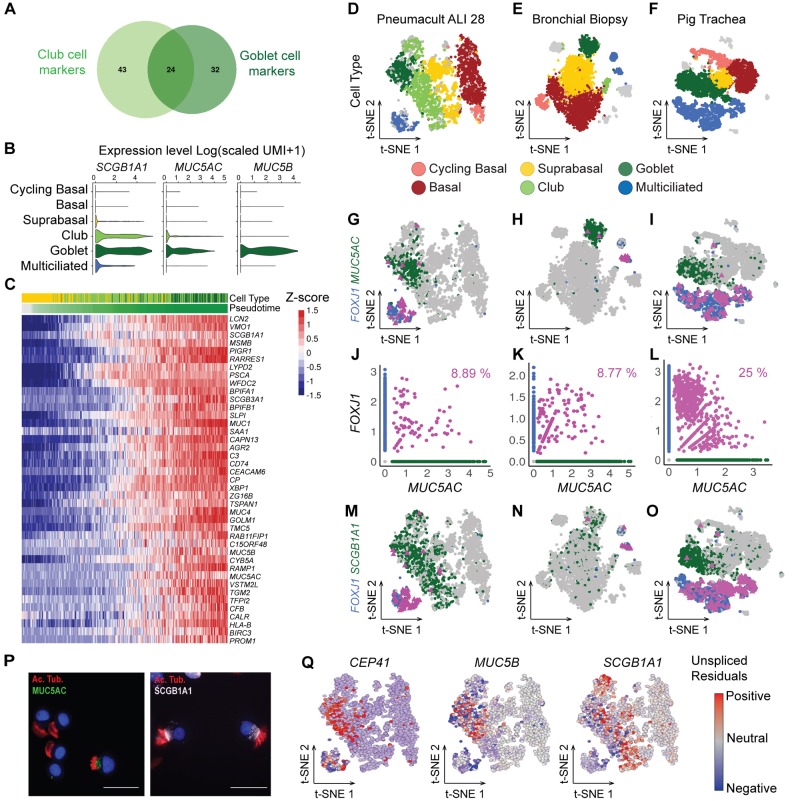
**Goblet cells as differentiation intermediates for multiciliated cells.** (A) Venn diagram illustrating the closeness of the best marker genes for club and goblet cells deduced from scRNA-seq of cells differentiated in Pneumacult medium (ALI 28). (B) Violin plots of normalized expression of *SCGB1A1*, *MUC5AC* and *MUC5B*, three markers of club and goblet cells. (C) Heatmap of the most differentially expressed genes between groups of suprabasal, club and goblet cells at key points in the pseudotime (before branching, start of the GC branch and end of the GC branch). Cells are ordered by pseudotime. Bars on the top of the heatmap indicate cell type and pseudotime. (D-F) t-SNE plots of expression from scRNA-seq of ALI 28 (D), bronchial biopsy cells (E) and newborn pig tracheal cells (F). (G-I) Highlights of gene expression for *FOXJ1*+ cells (blue), *MUC5AC*+ cells (green) and *FOXJ1*+*/MUC5AC*+ cells (pink) in the same samples as in D-F. (J-L) Relationships between normalized expression of *MUC5AC* and *FOXJ1* in the three same samples. (M-O) Highlights of gene expressions for *FOXJ1*+ cells (blue), *SCGB1A1*+ cells (green) and *FOXJ1*+/*SCGB1A1*+ cells (pink). (P) Immunodetection of cells co-expressing markers of multiciliated cells (acetylated tubulin) and of goblet cells (MUC5AC) (left) or of club cells (SCGB1A1) (right). Scale bars: 50 µm. (Q) Representation by a t-SNE plot (scRNA-seq of cells differentiated in Pneumacult medium at ALI 28) of the RNA velocity residuals colored according to estimates of the positive (red) and negative (blue) residuals for a multiciliated cell marker (*CEP41*), a goblet cell marker (*MUC5B*) and a club cell marker (*SCGB1A1*).

We further tested the hypothesis that some GCs correspond to MCC precursors. In clustering analyses, either from fresh tissues or from *in vitro* samples, GC and CC populations displayed very similar gene expression profiles, being discriminated by higher *MUC5AC* and *MUC5B* expression levels in GCs (Table S1). In Pneumacult, 24 of the 54 top genes for GCs were also associated with CCs (Fig. 2A), including *SCGB1A1*. Expression of *MUC5AC* and *MUC5B* was stronger in GCs (Fig. 2B). A direct assessment of differential gene expression between cells located at the two ends of the GC branch confirmed the high similarity of gene expression existing between CCs and GCs (Fig. 2C; Table S3A,B). GCs differed from CCs by higher levels of mucins (*MUC1*, *MUC4*, *MUC5B* and *MUC5AC*), secretoglobins (*SCGB1B1* and *SCGB3A1*), PLUNC antimicrobial factors (*BPIFA1* and *BPIFB1*) and *SLPI*, the secretory leukocyte protease inhibitor (Fig. 2C). These properties led us to consider GCs as ‘hyperactive’ CCs and led to the prediction that these cells could also function as MCC precursors. This point was tested by quantifying the expression of *MUC5AC* and *FOXJ1*, and by measuring the percentage of double-labeled cells. Detecting cells simultaneously expressing *MUC5AC* and *FOXJ1* would suggest the existence of a transitory state between GCs and MCCs. Fig. 2D,G,J indeed shows that 8.9% of GCs and MCCs simultaneously express *MUC5AC* and *FOXJ1*. It also shows the existence of CCs/MCCs expressing both *SCGB1A1* and *FOXJ1*, which correspond to a more conventional type of precursor for MCCs (Fig. 2M). The presence of *MUC5AC*+/*FOXJ1*+ and *SCBG1A1*+/*FOXJ1*+ cells was not restricted to a cell culture differentiation model, and these transitionary cells were also detected in fresh biopsies from human homeostatic bronchi (Fig. 2E,H,K,N) and newborn pig trachea (Fig. 2F,I,L,O).

Hybrid cells were also detected by qRT-PCR in a fully independent HAEC culture, after isolation of the cells using C1 technology (Fluidigm) and quantification of gene expression with a Biomark (Fluidigm). Cells isolated with the C1 were visually inspected, and these experimental settings ensured the absence of cell doublets. Four cells out of 74 expressed GC-specific genes (namely *MUC5AC*, *MUC5B* and *TFF3*), together with MCC-specific genes (*FOXJ1*), and more specifically, immature MCC genes (*PLK4*, *MYB* and *CDC20B*) (Revinski et al., 2018) (Fig. S7A,B). This result was confirmed after re-analyzing a recently published dataset (Plasschaert et al., 2018) (Fig. S7C,D). A further confirmation came from the detection at the protein level of cells that were simultaneously labeled for MUC5AC and acetylated tubulin, a specific protein marker of the cilia (Fig. 2P). A final point came after a survey of our data with two additional algorithms: ‘RNA velocity’ (La Manno et al., 2018) and Palantir (Setty et al., 2019). RNA velocity can predict the fate of individual cells over a timescale of hours by distinguishing the expression of spliced and unspliced forms of transcripts. We analyzed with RNA velocity the behavior of *CEP41*, *SCGB1A1* and *MUC5B*, in which *CEP41* is an early marker of multiciliated cells differentiation. RNA velocity calculates a residual value of each gene, which indicates expected upregulation when it is positive and expected downregulation when it is negative. Positive residuals were found for transcripts of *CEP41* in the GC population, predicting an upregulation of *CEP41* over the following hours. A different picture was observed for the transcripts of *SCGB1A1* and *MUC5B*, in which negative residuals were found in the GC and CC populations, indicating an expected downregulation of the corresponding transcripts over the following hours (Fig. 2Q). We then explored the same dataset with Palantir, another algorithm that models cell trajectory, with which we confirmed the presence of GCs on the MCC branch (Fig. S7E). The score for differentiation potential was highest for cycling basal cells. A high score was also found in the MCC branch in a region containing both CCs and GCs, before the gap separating them from MCCs (Fig. S7F), further suggesting a high probability to differentiate into at least two distinct trajectories. Estimation of gene expression trends showed an upregulation and then a downregulation of both MUC5AC and MUC5B along the pseudotime in cells committed to the MCC lineage (Fig. S7G). Finally, computing branch probabilities of randomly selected GCs on the MCC branch showed that some of them have between 24.7% and 49.7% chance of following the MCC trajectory (Fig. S7H). Altogether, these data indicate that GCs can act as precursors for MCCs in normal *in vitro* and in homeostatic *in vivo* airway regeneration.

### Refining cell clustering identifies six additional clusters, including a discrete population of pre-MCC ‘deuterosomal’ cells

**Fig. 3.**
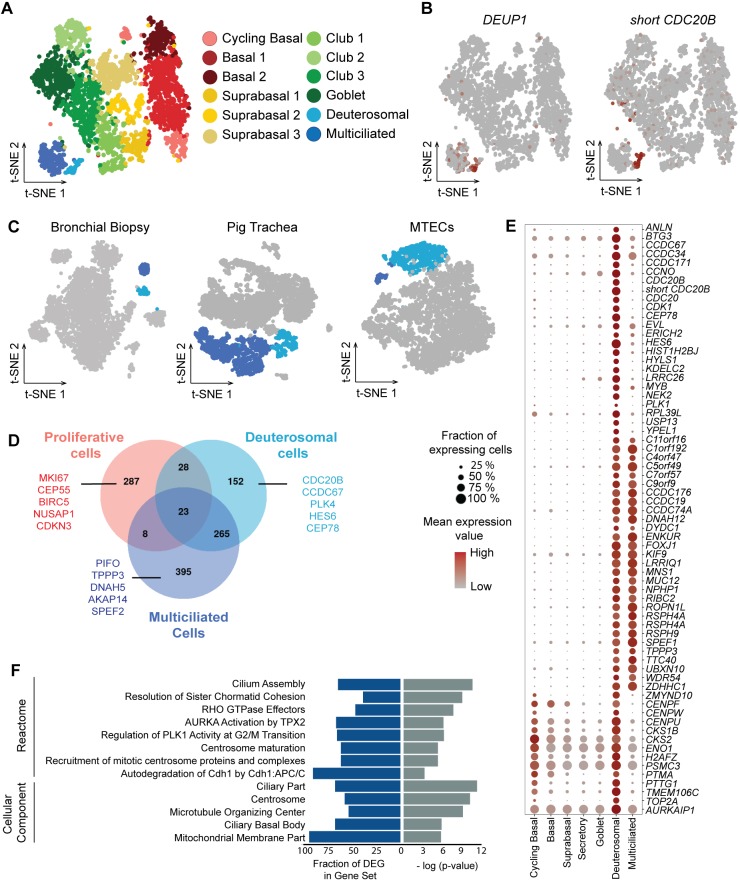
**Deuterosomal cells form a discrete multiciliated cell intermediate population with a centriole amplification signature.** (A) Subclusterization of scRNA-seq from cells differentiated in Pneumacult medium (ALI 28) into 12 cell types, deduced from intra-heterogeneity analysis of the six initial clusters. (B) Illustration of the specific expression of *DEUP1* and *short CDC20B* in the deuterosomal cell population (low to high expression, gray to red). (C) Identification of the cluster of deuterosomal cells in scRNA-seq data from a biopsy of human bronchi, newborn pig trachea and mouse primary culture (MTEC, ALI 3, stage of higher centriole amplification). Light blue, deuterosomal cells; dark blue, multiciliated cells. (D) Venn diagram showing that overlaps exist between top gene markers of deuterosomal cells (light blue) and those of proliferative (pink) or multiciliated cells (dark blue). (E) Dot plot of marker genes for the deuterosomal cell population. Color gradient (gray to red) and dot size indicate for each cluster the mean marker expression and the percentage of cells expressing the marker, respectively. (F) Enriched gene sets in deuterosomal cell marker genes.

To gain further insight into the diversity of cell populations composing the airway epithelium and the transitionary cell populations occurring during the regeneration, we considered additional clusters that could be derived from our sub-clustering analysis, by accepting less discriminations between them than between the six previously identified clusters. This deeper analysis led to the identification of 12 clusters, instead of six (Fig. 3A; Fig. S8A and Table S4). The non-cycling BC population was split into two clusters that we termed BC1 and BC2. The major difference between these two clusters was the higher level of expression of genes associated with cell migration: *FN1*, *VIM*, *SPARC* and *TAGLN* in the BC2 cluster. Analysis of enriched canonical pathways with Ingenuity Pathway Analysis showed enrichment for integrin, actin cytoskeleton and Rho GTPase signaling, as well as the pathway ‘regulation of actin-based motility’ in BC2 compared with BC1, suggesting an increased migratory activity in BC2 (Fig. S9). The supraBC and CC populations could also be further split into three new populations of supraBC and three new populations of CCs (Fig. 3A; Fig. S8A). Each of them displayed its own distinct gene set enrichment (Fig. S9). The CC2 subpopulation displayed a strong enrichment score for the feature ‘immune cell migration, invasion and chemotaxis’, and a strong positive enrichment for canonical pathways such as ‘neuroinflammation signaling’ and ‘dendritic cell maturation’. This was explained by an increased gene expression of targets for pro-inflammatory molecules such as TNF, IFNG, NFkB, IL1A/B, IL2 or IL6, as well as decreased gene expression for targets for the anti-inflammatory PPARG pathway (Fig. S9). This may confer to this subpopulation of CCs a unique relationship with the immune response. This subpopulation was confirmed in nasal and bronchial epithelia in a subset of healthy subjects from a Human Cell Atlas cohort (data not shown).

The MCC group of *FOXJ1+* cells was further split in two discrete clusters: (1) the largest one is positive for mature MCC genes such as *DNAH5*, and corresponds to terminally differentiated MCCs; (2) the second one specifically expresses several molecules that are important for the biosynthesis of hundreds of basal bodies from which motile cilia elongate. Among them is *DEUP1*, a hallmark of massive centriole amplification at deuterosomes (Fig. 3B). We named these cells ‘deuterosomal’ cells. This subpopulation is clearly distinct from mature MCCs (Fig. 3B) and expresses highly specific markers such as *PLK4*, *CCNO* and *CEP78* (Fig. S10A and Table S5A-C). Existence of deuterosomal cells was confirmed in mouse tracheal epithelial cells (MTECs) dissociated at ALI 3, in newborn pig trachea and in human bronchial biopsy tissue (Fig. 3C; Fig. S10B,C). All samples, even under homeostatic conditions, displayed deuterosomal cells that clustered independently of mature MCCs. In adult mouse trachea, we detected Deup1+ cells by immunohistochemistry that were clearly distinct from mature MCCs (multiple centrioles but no cilia). MCCs were devoid of Deup1 protein (Fig. S10D). Deuterosomal cells expressed unique gene markers, but also genes found in MCCs and cycling BCs (Fig. 3D). Our analysis found 149 specific genes, and 33 and 244 genes shared with cycling BCs and mature MCCs, respectively (Fig. 3E; Table S5). Among the 33 genes in common with cycling BCs, we noticed the re-expression of several cell cycle-related genes, which are required for the massive amplification of centrioles that takes place (Al Jord et al., 2017; Revinski et al., 2018). The most specific genes are displayed in Fig. 3E. This analysis not only confirms the known expression of *CDK1* in deuterosomal cells (Al Jord et al., 2017), it also highlights the expression in deuterosomal cells of genes coding for centromere proteins (*CENPF*, *CENPU* and *CENPW*), securin (*PTTG1*), a core subunit of the condensing complex (*SMC4*) and cyclin-dependent kinase regulatory subunits (*CKS1B* and *CKS2*). We confirmed the deuterosomal-specific expression of *CDC20B*, the miR-449 host gene that we have recently shown to be a key regulator of centriole amplification by deuterosomes (Revinski et al., 2018). Incidentally, a splice variant of this gene was detected, including a novel exon near the location of the *miR-449* family (Fig. 3B; Fig. S11A). This short *CDC20B* isoform was also detectable in mouse RNA-seq data (Fig. S11B). Comparison of transcript abundance in several samples, including the Pneumacult ALI 28 and the human bronchial biopsy tissue, showed higher levels for short *CDC20B* (Fig. S11C,D), which likely corresponds to the major source of *miR-449* in deuterosomal cells. A list of novel markers of deuterosomal cells that are specifically expressed in this cell population is provided in Table S5. Some of these genes have never been described before in the context of centriole amplification, such as the yippee-like factor *YPEL1* or the Notch pathway-related hairy-enhancer-of-split family of transcription factors *HES6* (Fig. S10A-C). Gene set enrichment of the deuterosomal population-specific genes (Fig. 3F) showed enrichments for ‘cilium assembly’ and ‘centrosome maturation’, but also cell-cycle mechanism-related terms such as ‘resolution of sister chromatid cohesion’, ‘regulation of AURKA’, ‘PLK1 activity’ and ‘CDH1 autodegradation’. ‘Mitochondrial membrane part’ was also among the enriched terms, suggesting an increase in mitochondria numbers at this stage. This signature perfectly delineates the events occurring at this MCC differentiation stage and provides an extensive repertoire of specific cell-cycle related genes that are re-expressed at the deuterosomal stage. The pool of deuterosomal cells was consistently larger than recently described rare cell populations such as ionocytes (Montoro et al., 2018; Plasschaert et al., 2018), which we also identified (Fig. S8C).

### Establishing a keratin switch pattern during airway regeneration

**Fig. 4.**
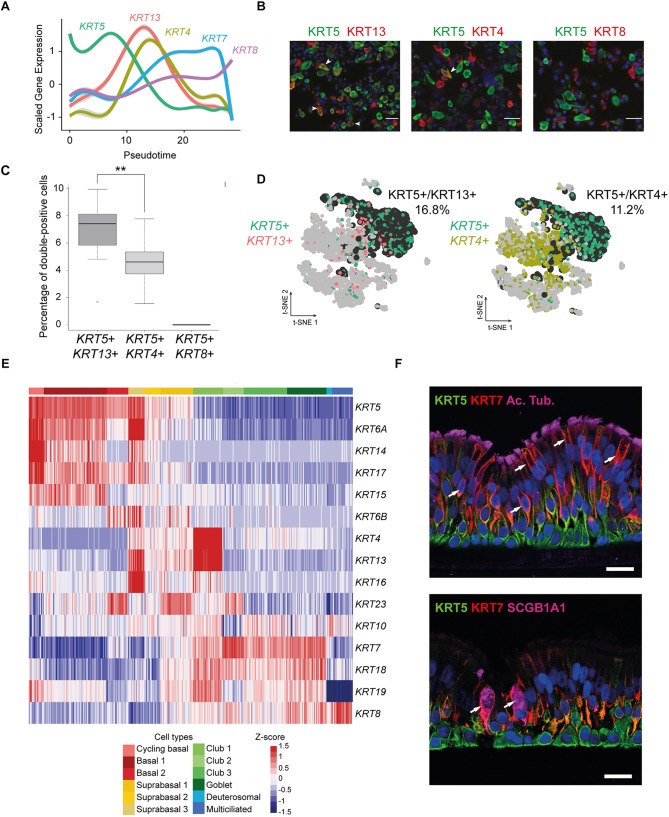
**Keratin signature switch during airway regeneration.** (A) Plot of normalized gene expression of keratins according to pseudotime from scRNA-seq of cells differentiated in Pneumacult medium (ALI 28). (B) Double immunofluorescence staining for KRT5 and KRT13, KRT4 or KRT8. White arrowheads indicate doubly labeled cells (KRT5+/KRT13+, KRT5+/KRT4+, KRT5+/KRT8+). Nuclei are shown in blue (DAPI). (C) Quantification of double-positive cells from B. **P<0.01 (Wilcoxon test). The black line inside each box represents the median. The vertical size of the boxes are the interquartile range, or IQR. Whiskers indicate 1.5×IQR for the box at the extreme left, or most extreme values in the other two boxes. (D) tSNEs of scRNA-seq data from pig tracheal epithelial cells. *KRT5+* cells are shown in emerald green, *KRT13+* cells are shown in red, *KRT4+* cells are shown in yellow-green and double-positive cells are shown in black. The indicated percentage corresponds to double-positive cells. (E) Heatmap for scRNA-seq data from Pneumacult ALI28 showing gene expression for keratins. (F) Immunohistochemistry for KRT5, KRT7 and acetylated tubulin or SCGB1A1 on sections of Pneumacult fully differentiated *in vitro* epithelium. Arrows indicate KRT7+ luminal non-multiciliated cells. Scale bars: 20 µm.

A rich repertoire of keratins is expressed in different epithelial cells, depending of cell type, period of embryonic development, stage of histological differentiation, cellular growth environment, disease state, etc. We screened our scRNA-seq data for expression of different keratins, besides *KRT5* and *KRT14*, which are bona fide BC markers in the airways and lung, but also in bladder (Colopy et al., 2014), prostate (Hudson et al., 2001) and mammary gland (Jumppanen et al., 2007), or for KRT8, which is clearly associated with luminal cell types (Rock et al., 2009). A recent study performed on mouse and human models of *in vitro* regeneration identified *KRT4* and *KRT13* in a subpopulation reminiscent of our supraBCs, as it emerges between BCs and CCs (Plasschaert et al., 2018). Our repertoire of KRT expression during airway regeneration was based on pseudotime ordering in our Pneumacult ALI 28 dataset. Our analysis confirmed the presence of *KRT5* and *KRT14* in BCs, of *KRT4* and *KRT13* in supraBCs, and the expression of *KRT8* in luminal cell types (CCs, GCs and MCCs) (Fig. 4A,E). Unlike recent data obtained by Plasschaert et al. under similar conditions (Plasschaert et al., 2018), who showed parallel RNA expression of *KRT13* and *KRT4*, we consistently noticed that expression profiles of *KRT13* and *KRT4* were slightly de-correlated, with *KRT13* detected at earlier pseudotimes than *KRT4*. This was confirmed at the protein level by a quantification of immunostainings of the proportion of KRT5+/KRT13+ and KRT5+/KRT4+ double-positive cells (Fig. 4B). Fig. 4C shows that there were more KRT5+/KRT13+ (7.4%) than KRT5+/KRT4+ (4.9%) double-positive cells, consistent with an earlier expression of KRT13 compared with KRT4. A similar observation was made in the newborn pig trachea, in which we also found a very clear shift, with 16.8% and 11.2% of *KRT5*+/*KRT13*+ and *KRT5*+/*KRT4*+ double-positive cells, respectively (Fig. 4D). Our results show that *KRT4* and *KRT13* are not strictly expressed at the same time during airway regeneration and their expression delineates subtle differences in cell subpopulations. In homeostatic nasal epithelium, we noticed an even greater uncoupling of KRT4 and KRT13 expression at RNA and protein levels. In scRNA-seq, KRT13 was highest in cycling BCs, then in BCs and supraBCs. KRT4 was highest in CCs, then in supraBCs and cycling BCs (Fig. S12A). Immunostaining on nasal turbinate epithelium confirmed that KRT13 was predominantly found at a basal position, and KRT4 at a luminal position (Fig. S12C). Hence, KRT4 and KRT13 cell-type specificity might differ according to the homeostatic or regenerative status. Additional keratins, such as *KRT16* and *KRT23* displayed a specific supraBC expression (Fig. 4E). We also identified additional keratins that were more specifically associated with differentiated cell types: *KRT7* and *KRT19* were strongly enriched in CCs, but their expression completely dropped in MCCs, while *KRT8* was still expressed (Fig. 4E). Expression patterns for these cell type-specific keratins were confirmed by immunohistochemistry on sections of ALI culture and nasal epithelium (Fig. 4F; Fig. S12B,D). Altogether, our data indicate that the keratin repertoire can be sufficiently specific to reconstruct cell trajectories during airway regeneration.

### Establishing a combinatorial repertoire of signaling pathways during airway regeneration

We have finally analyzed the cell specificity of expression of important signaling pathways in order to determine mutual influences between distinct cells that could play a role in airway regeneration. Our investigation was focused on the Notch, BMP/TGFβ and Wnt pathways. For each different component, we classified them as ligands, receptors, or targets. The expression profiles are shown as heatmaps, with cells being sorted by their subgroups.

#### Notch pathway

**Fig. 5.**
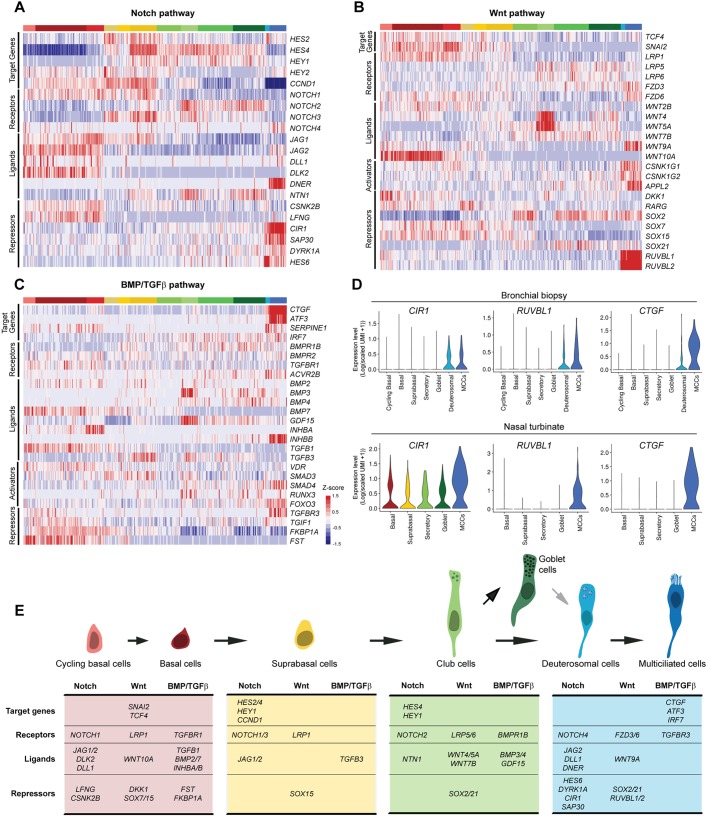
**Single-cell expression of signaling pathway components during airway regeneration.** (A) Heatmap of the genes related to the Notch pathway with cells ordered by clusters. (B) Heatmap of the genes related to the Wnt pathway with cells ordered by cluster. (C) Heatmap of the genes related to the BMP/TGFβ pathway with cells ordered by cluster. (D) Violin plots for selected genes in the bronchial biopsy and nasal turbinate samples. (E) Summary of the major partners involved in specific cell types for the three pathways.

BCs express the ligands DLL1, JAG1 and JAG2, as well as the receptor NOTCH1, as expected (Plasschaert et al., 2018; Rock et al., 2009). In this population, no target gene expression was detected, suggesting an inactive pathway. BCs also express *LFNG*, which is known to inhibit JAG1 signaling via NOTCH1 (Yang et al., 2004). SupraBCs cells express *NOTCH1*, *JAG1* and *JAG2*, and show clear activation of the Notch pathway by expression of the target genes *HEY1*, *HES2* and *HES4*. *NOTCH3* expression is turned on and is specific to this population. In CCs/GCs, NOTCH2 is the major receptor to be detected and signal activation remains, as evidenced by the expression of *HEY1* and *HES4*. CCs/GCs also express the non-canonical Notch ligand *NTN1*. In deuterosomal cells/MCCs, a clear shift is observed. Expression of *NOTCH2*, *NOTCH3*, *HEY1* and *HES4* is reduced, and *NOTCH4* is specifically expressed. As previously described, *JAG2* (Plasschaert et al., 2018), which is present in BCs then absent in supraBCs and CCs/BCs, is re-expressed in the MCC compartment. We have found the same behavior for *DLL1* and the non-canonical ligand *DNER*. Thus, MCC express some Notch ligands. Strikingly, a major inhibitory signature dominates in MCCs, with the expression of *CIR1* and *SAP30*, two transcriptional co-repressors, and of *DYRK1A*, an inhibitor of the NICD. *HES6*, the expression of which is not regulated by Notch signaling but has been identified as a Notch pathway inhibitor (Bae et al., 2000), is highly enriched in deuterosomal cells (Figs 5A and 3E). We have confirmed at the protein level an enrichment of SAP30 in MCCs (Fig. S13A).

#### Wnt pathway

The Wnt target genes *SNAI2* and *TCF4*, which are indicators of an active pathway, are mainly enriched in the BC population, especially in BC2 for SNAI2. We have confirmed enrichment of SNAI2 in BCs at the protein level (Fig. S13B). In the BC population, *WNT10A* and *LRP1* are strongly enriched, and several SOX family members (*SOX2* and *SOX21*) are underrepresented, especially in the cycling BCs, suggesting an activation of the pathway in this compartment. In the MCC population, the situation is more complex. Despite the slight expression of *TCF4* together with positive regulators of the pathway, such as *WNT9A*, *FZD6*, *APPL2*, *CSNK1G1* (a casein kinase component that can act as an activator or inhibitor of the pathway; Cruciat, 2014), no *SNAI2* expression is detected, and known repressors of the Wnt pathway are also overrepresented. Indeed, MCCs express significant levels of the transcriptional repressors *SOX2* and *SOX21*, and display strong enrichment for the reptin components *RUVBL1* and *RUVBL2* (Fig. 5B).

#### BMP/TGFβ

BMP ligands, such as *BMP2* and *BMP7*, are enriched in the BC population, while *BMP3* and *BMP4* are both enriched in the CC/GC populations. We did not find any specific cell population expression for BMP receptors. Specific expression of *FST* (follistatin) and *FKBP1A* (also known as *FKBP12*), two BMP inhibitors, was found in BCs, which was confirmed for FST in BCs at the protein level (Fig. S13C,D). Regarding the TGFβ pathway, a clear signal of activation is detected in the deuterosomal/MCC population, with specific expression of the target genes *SERPINE1* (PAI-1), *CTGF*, *ATF3*, *TGFBR3* and *IRF7*, consistent with the previous finding that TFGβ pathway regulates motile cilia length by affecting the transition zone of the cilium (Tözser et al., 2015). We did not detect TGFβ ligands in the MCC population but rather found them expressed in BCs (TGFB1) and supraBCs (TGFB3).

We have confirmed the main distribution of the three pathway components in samples differentiated with the BEGM medium (Fig. S14) and in two fresh tissue samples (human bronchial biopsy and nasal turbinate) for which a selection of genes is shown in Fig. 5D. Collectively, our data provide for the first time a detailed account of Notch, Wnt and BMP signaling pathways at work during airway regeneration, with receptors and ligands specifically expressed at each cell stage.

## DISCUSSION

We have established here a comprehensive single-cell atlas throughout the entire time course of human nasal airway differentiation *in vitro*. We quantified the proportion and identity of each cell population at carefully chosen time points after the establishment of the air liquid interface. We provide the first comparison between the most widely used culture media in the 3D culture of airway epithelial cells, BEGM (with which the majority of studies have been performed), and a more recently available commercial medium, Pneumacult. In the BEGM medium, we have performed analyses at earlier time points, i.e. ALI 2 and ALI 4. These time points allowed us to measure the extent of cell proliferation during *in vitro* regeneration. Cycling BCs accounted for ∼40% of total cells at ALI 2 and ALI 4, and this number dropped to 5% at ALI 7. These early time points also showed that supraBCs appeared early under these conditions, being already detected at ALI 4. With BEGM, we never detected any GCs (*MUC5AC*+) or ‘canonical’ CCs (*SCGB1A1*+), even after long periods of time and using several dozens of cultures from distinct donors (Figs S1, S3, S4; data not shown). However, we found a cell population that we have termed ‘club-like’. These ‘club-like’ cells express a gene pattern very similar to that of canonical CCs, and they can differentiate into MCCs. Interestingly, GCs were detected in BEGM medium after IL13 treatment (Laoukili et al., 2001; data not shown). Future work should investigate whether club-like cells first evolve into canonical CCs and then GCs upon IL13 treatment.

In Pneumacult, but also in freshly dissociated human bronchial biopsy tissue and newborn pig trachea, we have detected hybrid cells expressing both *MUC5AC* and *FOXJ1*. This finding is consistent with our lineage inference, as RNA velocity and Palantir analyses consistently defined GCs as possible precursors of multiciliated cells. Other groups have previously detected cells expressing both markers, in a context of GC hyper/metaplasia induced by Sendai virus infection or after IL13 treatment and in asthma (Gomperts et al., 2007; Turner et al., 2011; Tyner et al., 2006; Vieira Braga et al., 2019). These findings led some of them to hypothesize a transdifferentiation of MCCs into GCs. However, no convincing data support this conclusion and none of these data show a difference in the number of these hybrid cells between control and treated conditions. For example, Turner and colleagues (Turner et al., 2011) postulated this after performing *in vitro* lentiviral transduction of HAECs with a vector containing a Cre recombinase under the control of the *FOXJ1* promoter. However, no control demonstrated the absence of leakage of the *FOXJ1* promoter and these findings were not confirmed by Rajagopal's group who showed no GCs arising from MCCs in a context of OVA-induced mucous metaplasia in mouse airways, using *in vivo* lineage tracing with *Foxj1*-cre mice (Pardo-Saganta et al., 2013). Our contribution to resolve this conundrum is by showing that these hybrid cells do exist in the absence of I-13 stimulation and in healthy subjects. We therefore suggest that their expression profiles place them more straightforwardly as alternative precursors of MCCs than as trans-differentiated MCCs.

As our work was performed on either cultured or fresh cells from nasal or lung airways derived from three distinct animal species, the generalization of some of our conclusions to mouse, human and pig airways is probably justified. This is probably the case for the general mechanisms of MCC and GC differentiations. At the same time, we are also aware of the important gradients of gene expression that exist between different compartments, as already documented between nose and bronchi (Giovannini-Chami et al., 2018). Future work will have to address the origins of these spatial idiosyncrasies. Our study was also not intended to characterize rare cell types such as pulmonary neuroendocrine, brush cells or ionocytes, which have recently been described elsewhere. We confirm the detection of cells displaying high levels of expression of *CFTR*, *ASCL3* and *FOXI1*, corresponding to pulmonary ionocytes (Montoro et al., 2018; Plasschaert et al., 2018). Our investigation was more focused on the main cell types that compose the epithelium, and their underlying mechanisms of differentiation. Three subtypes of BCs were identified, including a group of cycling BCs, and a group of BCs expressing higher levels of genes involved in extracellular matrix connection and actin-based motility. This latter group is reminiscent of that described by Coraux et al. who showed that airway BCs undergo changes in the cytoskeleton organization and acquire mesenchymal cell-associated vimentin as well as various matrix metalloproteinases necessary for migration above the denuded basement membrane in response to injury (Coraux et al., 2008). This BC subtype is probably specific to regeneration and should not be detected in homeostatic samples. Accordingly, few such cells were found in nasal and bronchial epithelial samples from 12 healthy subjects of the Human Cell Atlas (data not shown).

The specificity of the secretory compartment comes from one club cell subpopulation that displayed an immune-related gene signature. So far, diversity within the club cell compartment is thought to be established after expression of different members of the secretoblogin family (Reynolds et al., 2002) or via an appropriate activation level of the Notch pathway (Guha et al., 2014). We propose that diversity within this cell compartment should also include specialized functions related to the interaction between the epithelium and immune cells. Additional experiments, including protein labeling on fresh tissue sections from several levels of the airways, have now to be performed in order to confirm this diversity and identify the spatial distribution of these subpopulations.

Our study has also provided a first extensive gene signature of the deuterosomal population, which plays a key role during MCC differentiation. This population comprises three to four times fewer cells than the MCC population, suggesting that each cell transits quickly through this stage. In line with what has been shown recently by our group and others (Al Jord et al., 2017; Revinski et al., 2018; Vladar et al., 2018), cell cycle-related genes become re-expressed in this population of non-cycling cells. We have confirmed the very specific expression of *CDC20B*, a key player of centriole amplification (Revinski et al., 2018), and have identified, both in human and mouse, a novel isoform of this transcript that displays higher expression than the annotated long isoform. As the pre-mRNA corresponding to this short isoform comprises the *miR-449-*encoding intron, we suggest that this isoform should indeed be the major source of *miR-449* in deuterosomal cells. The alternative splicing that is responsible for this alternative isoform might represent an optimization of gene expression regulation to efficiently increase *miR-449* levels.

We also characterized the distribution of important signaling pathways. We started with the Notch pathway as it is a major regulator of the mucociliary differentiation. We have confirmed the distribution of ligands and receptors described by others (Mori et al., 2015; Pardo-Saganta et al., 2015b; Plasschaert et al., 2018; Rock et al., 2011). Absence of *HES4* expression, the most representative target gene in our model, confirmed the absence of Notch activation in BCs and MCCs. BCs rather express *NOTCH1* and NOTCH ligands. However, no clear Notch pathway activation can be detected within this cell population even in a patchy manner as might be expected from Notch lateral inhibition. This absence of activation might result from the weak *NOTCH1* expression or the expression of Notch inhibitors such as the ligand *LFNG* or casein kinase II subunit beta (*CSNK2B*) (Cheng et al., 2014; Wang et al., 2014). Inhibition of the Notch pathway in MCCs at the end of multiciliogenesis has been widely documented. Here, the specific expression of several Notch transcriptional inhibitors at the deuterosomal stage suggest a novel mechanism for this inactivation. This is the case for *HES6*, an inhibitory HES acting through HES1 binding (Bae et al., 2000; Nam et al., 2016), *DYRK1A*, an inhibitor of Notch intracellular domain transcriptional activity (Fernandez-Martinez et al., 2009), as well as *CIR1* and *SAP30*, which are transcriptional repressors of the Notch/CSL transcriptional complex (Hsieh et al., 1999). On the other hand, CCs must undergo clear Notch activation to maintain cell identity and differentiate into GCs (Pardo-Saganta et al., 2015b; Rock et al., 2011; Tsao et al., 2009). However, the onset of activation of this signal has not been widely studied. Mori and colleagues have described NOTCH3 expression in TP63-negative cells in a parabasal position of the epithelium, which likely correspond to the cells that we and others have termed supraBCs (Mori et al., 2015). We have confirmed that the *NOTCH3* transcript is absent from BCs and becomes upregulated in supraBCs. We went further by showing that *HES4* becomes expressed at this cell stage, confirming that Notch pathway activation starts at the supraBC stage. We emphasize here the importance of this intermediate cell population for establishing Notch activation and subsequent differentiation, even though it has not been well characterized so far.

The Wnt/β-catenin pathway has been less extensively studied in the context of airway epithelium differentiation (Brechbuhl et al., 2011; Malleske et al., 2018; Schmid et al., 2017; Smith et al., 2012; Zemke et al., 2009). Crosstalk with Notch has been suggested in non-airway studies: in hair follicle precortex, β-catenin stimulates Notch signaling by inducing *Jag1* transcription (Estrach et al., 2006). In the airway epithelium, β-catenin signaling is required at ‘specification’, i.e. early stages of GC and MCC differentiation, but was detrimental at later stages (Malleske et al., 2018). Ordovas-Montanes et al. have recently shown that Wnt is also related to inflammatory-induced epithelial remodeling. In nasal polyps, an imbalance between Wnt and Notch signaling favors Wnt signaling and GCs at the expense of MCCs (Ordovas-Montanes et al., 2018). In airway smooth muscle cells, *WNT5A* is associated with remodeling in a context of airway hyperresponsiveness (Koopmans et al., 2016). In HAECs from individuals with chronic obstructive pulmonary disease, *WNT4* upregulation increases *IL8* and *CXCL8* gene expression (Durham et al., 2013). Interestingly, *WNT5A* and *WNT4* were specifically expressed by the subpopulation of CCs related to immune response. This finding further reinforces the hypothesis of a role for this CC population in the inflammation-induced airway remodeling.

Based on expression of the target genes *TCF4* and *SNAI2*, activation of the Wnt pathway is confined to the BC population. *SNAI2* enrichment in the basal cell compartment had already been noticed by Rock and colleagues upon sorting of basal cells from mouse trachea (Rock et al., 2009). This population also strongly and specifically expresses the ligand *WNT10A*, suggesting an autocrine regulatory loop. WNT10A is also BC specific in other epithelia, such as the mammary epithelium (Ji et al., 2011). In fallopian organoids, Wnt has been shown to be essential for stemness (Kessler et al., 2015) and for self-renewal, but not for proliferation, in basal-like breast cancer cells (DiMeo et al., 2009). Thus, autocrine WNT10A signaling may also regulate self-renewal in the BC compartment of the airway epithelium. In contrast, we have observed in MCCs a specific expression of the two ATP-dependent DNA helicases from the Reptin family that act as Wnt signaling repressors (Bauer et al., 2000; Weiske and Huber, 2005). Additional investigations should certainly be carried out to characterize more precisely the role of Wnt/β-catenin during airway epithelial regeneration.

Regarding the TGFβ/BMP pathway, our data strongly suggest inhibition of this pathway in the BC compartment. As this signaling is considered to be a brake for proliferation, our findings are consistent with a previous report showing maintenance of a proliferative potential of this progenitor population by dual SMAD inhibition (Mou et al., 2016).

## Conclusions

We provide several novel insights in the dynamics of airway differentiation by positioning goblet cells as possible precursors of multiciliated cells: this illustrates how cells carrying specialized function, i.e. club and goblet cells, can still constitute differentiation intermediates for other specialized cells, i.e. multiciliated cells. We also identify subpopulations of basal, suprabasal, club and multiciliated cells. Our dataset also provides extensive characterization of the deuterosomal cell population, an intermediate state before the formation of multiciliated cells. After establishing a comprehensive repertoire of keratin expression, we show that monitoring ‘keratin switch’ during differentiation could be self-sufficient to establish the different cell identities. Our improved characterization of the different signaling pathway components detects putative Notch repressors that probably contribute to Notch signal shutdown at the deuterosomal stage, and details Wnt pathway activity within the basal cell compartment.

## MATERIALS AND METHODS

### Human airway epithelial cell culture

Human airway epithelial cell (HAEC) cultures were derived from nasal mucosa of inferior turbinates. After excision, nasal inferior turbinates were immediately immersed in Ca^2+^/Mg^2+^-free HBSS supplemented with 25 mM HEPES, 200 U/ml penicillin, 200 µg/ml streptomycin, 50 µg/ml gentamicin sulfate and 2.5 µg/ml amphotericin B (all reagents from Gibco). After repeated washes with ice-cold supplemented HBSS, tissues were digested with 0.1% Protease XIV from *Streptomyces griseus* (Sigma-Aldrich) overnight at 4°C. After incubation, fetal calf serum (FCS) was added to a final concentration of 10%, and nasal epithelial cells were detached from the stroma by gentle agitation. Cell suspensions were further dissociated by trituration through a 21 G needle and then centrifuged at 150 ***g*** for 5 min. The pellet was resuspended in supplemented HBSS containing 10% FCS and centrifuged again. The second cell pellet was then suspended in Dulbecco's Modified Eagle's Medium (DMEM, Gibco) containing 10% FCS and cells were plated (20,000 cells per cm^2^) on 75 cm^2^ flasks coated with rat-tail collagen I (Sigma-Aldrich). Cells were incubated in a humidified atmosphere of 5% CO_2_ at 37°C. Culture medium was replaced with bronchial epithelium basal medium (BEBM, Lonza) supplemented with BEGM SingleQuot Kit Supplements (Lonza) on the following day and was then changed every other day. After 4 to 5 days of culture, after reaching about 70% confluence, cells were detached with trypsin-EDTA 0.05% (Gibco) for 5 min and seeded on Transwell permeable supports (6.5 mm diameter; 0.4 µm pore size; Corning) in BEGM medium at a density of 30,000 cells per Transwell. Once the cells have reached confluence (typically after 5 days), they were induced to differentiate at the air-liquid interface by removing medium at the apical side of the Transwell, and by replacing medium at the basal side with either DMEM:BEBM (1:1) supplemented with BEGM SingleQuot Kit Supplements or with Pneumacult-ALI (StemCell Technologies), as indicated in the figure legends. Culture medium was changed every other day.

### Mouse tracheal epithelial cell culture

Mouse tracheal epithelial cell (MTEC) cultures were established from the tracheas of 12-week-old C57BL/6 mice. After dissection, tracheas were placed in ice-cold DMEM:F-12 medium (1:1) supplemented with 15 mM HEPES, 100 U/ml penicillin, 100 µg/ml streptomycin, 50 µg/ml gentamicin sulfate and 2.5 µg/ml amphotericin B. Each trachea was processed under a binocular microscope to remove as much conjunctive tissue as possible with small forceps and was opened longitudinally with small dissecting scissors. Tracheas were then placed in supplemented DMEM:F-12 containing 0.15% protease XIV from *S. griseus*. After overnight incubation at 4°C, FCS was added to a final concentration of 10%, and tracheal epithelial cells were detached by gentle agitation. Cells were centrifuged at 400 ***g*** for 10 min and resuspended in supplemented DMEM:F-12 containing 10% FCS. Cells were plated on regular cell culture plates and maintained in a humidified atmosphere of 5% CO_2_ at 37°C for 4 h to allow attachment of putative contaminating fibroblast. Medium-containing cells in suspension were further centrifuged at 400 ***g*** for 5 min and cells were resuspended in supplemented DMEM:F-12 containing BEGM Singlequot kit supplements and 5% FCS. Cells were plated on rat tail collagen I-coated Transwell. Typically, five tracheas resulted in 12 Transwells. Medium was changed every other day. Air-liquid interface culture was conducted once transepithelial electrical resistance had reached a minimum of 1000 Ω/cm^2^ (measured with EVOM2, World Precision Instruments). Air-liquid interface culture was obtained by removing medium at the apical side of the Transwell and by replacing medium at the basal side with Pneumacult-ALI medium (StemCell Technologies).

### HAEC and MTEC dissociation for single-cell RNA-seq

Single-cell analysis was performed at the indicated days of culture at the air-liquid interface. To obtain a single-cell suspension, cells were incubated with 0.1% protease type XIV from *S. griseus* (Sigma-Aldrich) in supplemented HBSS for 4 h at 4°C. Cells were gently detached from Transwells by pipetting and then transferred to a microtube. Fifty units of DNase I (EN0523 Thermo Fisher Scientific) per 250 µl were directly added and cells were further incubated at room temperature for 10 min. Cells were centrifuged (150 ***g*** for 5 min) and resuspended in 500 µl supplemented HBSS containing 10% FCS, centrifuged again (150 ***g*** for 5 min) and resuspended in 500 µl HBSS before being mechanically dissociated through a 26 G syringe (four times). Finally, cell suspensions were filtered through a 40 µm porosity Flowmi Cell Strainer (Bel-Art), centrifuged (150 ***g*** for 5 min) and resuspended in 500 µl of ice-cold HBSS. Cell concentration measurements were performed with a Scepter 2.0 Cell Counter (Millipore) and Countess automated cell counter (Thermo Fisher Scientific). Cell viability was checked with a Countess automated cell counter (Thermo Fisher Scientific). All steps except the DNAse I incubation were performed on ice. For cell capture using the 10× genomics device, the cell concentration was adjusted to 300 cells/µl in HBSS, aiming to capture 1500 cells for HAECs and 5000 cells for MTECs.

### Turbinate epithelial cell dissociation

To obtain a single-cell suspension directly from turbinates, the whole turbinate from a 30-year-old female donor was incubated with 0.1% protease type XIV from *S. griseus* (Sigma-Aldrich) in supplemented HBSS at 4°C overnight. Epithelial cells were gently detached from the turbinate by washing with HBSS by pipetting up and down, and then transferred to a 50 ml Falcon tube. Cells were centrifuged (150 ***g*** for 5 min at 4°C) and after removing the supernatant the cells were resuspended in 1 ml of HBSS. Fifty units of DNase I (EN0523 Thermo Fisher Scientific) per 250 µl were directly added and cells were further incubated at room temperature for 10 min. Cells were centrifuged (150 ***g*** for 5 min at 4°C) and resuspended in 1 ml supplemented HBSS containing 10% FCS, centrifuged again (150 ***g*** for 5 min at 4°C) and resuspended in 500 µl HBSS before being mechanically dissociated through a 26 G syringe (four times). Finally, cell suspensions were filtered through a 40 µm porosity Flowmi Cell Strainer (Bel-Art), centrifuged (150 ***g*** for 5 min) and resuspended in 500 µl of ice-cold HBSS. Cell concentration measurements were performed using a Scepter 2.0 Cell Counter (Millipore) and Countess automated cell counter (Thermo Fisher Scientific). Cell viability was checked with a Countess automated cell counter (Thermo Fisher Scientific). All steps, except the DNAse I incubation, were performed on ice. For the cell capture using the 10× genomics device, the cell concentration was adjusted to 500 cells/µl in HBSS aiming to capture 5000 cells.

### Anesthetic procedure

Intranasal anesthesia is performed with topical application (gauze) of 5% lidocaine (anesthetic) plus naphazoline (vasoconstrictor) solution (0.2 mg/ml). Laryngeal and endobronchial anesthesia is performed with topical application of 2% lidocaine through the working channel of a 4.9 mm outer diameter bronchoscope.

### Nasal brushing

Brushing was performed with a 2 mm cytology brush (Medi-Globe) in the inferior turbinate zone of a 56-year-old healthy male donor.

### Bronchial biopsy

Bronchial biopsy was performed at the spur between the left upper lobe and the left lower lobe with a 1.8 mm-diameter Flexibite biopsy forceps (Medi-Globe) passed through the working channel of the bronchoscope (WCB) on a 59-year-old male donor.

### Dissociation of nasal brushing

The brush was soaked in a 5 ml Eppendorf containing 1 ml of dissociation buffer, which was composed of HypoThermosol (BioLife Solutions), 10 mg/ml protease from *Bacillus Licheniformis* (Sigma-Aldrich, P5380) and 0.5 mM EDTA (Adam et al., 2017). The tube was shaken vigorously and centrifuged for 2 min at 150 ***g***. The brush was removed, cells pipetted up and down five times and then incubated cells on ice for 30 min, with gentle trituration with 21 G needles five times every 5 min. Protease was inactivated by adding 200 μl of HBSS/2% BSA. Cells were centrifuged (400 ***g*** for 5 min at 4°C). Supernatant was discarded leaving 10 μl of residual liquid on the pellet. Cells were resuspended in 500 μl of wash buffer (HBSS/0.05% BSA) and 2.25 ml of ammonium chloride 0.8% was added to perform red blood cell lysis. After a 10 min incubation, 2 ml of wash buffer was added and cells were centrifuged (400 ***g*** for 5 min at 4°C). Supernatant was discarded leaving 10 μl of residual liquid on the pellet, cells were resuspended in 1 ml of wash buffer and centrifuged (400 ***g*** for 5 min at 4°C). Supernatant was discarded leaving 10 μl of residual liquid on the pellet, cells were resuspended in 1 ml of wash buffer and passed through a 40 µm porosity Flowmi™ Cell Strainer (Bel-Art) then centrifuged (400 ***g*** for 5 min at 4°C). Supernatant was discarded, leaving 10 μl of residual liquid on the pellet. Cells were resuspended in 100 μl of wash buffer. Cell counts and viability were performed with a Countess automated cell counter (Thermo Fisher Scientific). For cell capture using the 10× genomics device, the cell concentration was adjusted to 500 cells/µl in HBSS, aiming to capture 5000 cells. All steps were performed on ice.

### Dissociation of bronchial biopsy

The biopsy tissue was soaked in 1 ml dissociation buffer, which was composed of DPBS, 10 mg/ml protease from *Bacillus licheniformis* (Sigma-Aldrich, P5380) and 0.5 mM EDTA. After 1 h, the biopsy was finely minced with a scalpel and returned to the dissociation buffer. From this point, the dissociation procedure is the same as the one described in the ‘dissociation of nasal brushing’ section, with an incubation time increased to 1 h, and omitting the red blood cell lysis procedure. For cell capture using the 10× genomics device, the cell concentration was adjusted to 300 cells/µl in HBSS, aiming to capture 5000 cells. All steps were performed on ice.

### Pig tracheal epithelial cell dissociation

To obtain a single-cell suspension from newborn pig trachea, whole clean tracheas were incubated with 0.1% protease type XIV from *S. griseus* (Sigma-Aldrich) in supplemented HBSS at 4°C overnight. Epithelial cells were gently detached from the turbinate by washing with HBSS and pipetting up and down, then transferring to a 50 ml Falcon tube. Cells were centrifuged (150 ***g*** for 5 min at 4°C) and after removing the supernatant the cells were resuspended in 1 ml of HBSS and 50 units of DNase I (EN0523, Thermo Fisher Scientific) per 250 µl were directly added. The cells were then further incubated at room temperature for 10 min. Cells were centrifuged (150 ***g*** for 5 min at 4°C) and resuspended in 1 ml supplemented HBSS containing 10% FCS, centrifuged again (150 ***g*** for 5 min at 4°C) and resuspended in 500 µl HBSS before being mechanically dissociated through a 26 G syringe (four times). Finally, cell suspensions were filtered through a 40 µm porosity Flowmi Cell Strainer (Bel-Art), centrifuged (150 ***g*** for 5 min) and resuspended in 500 µl of ice-cold HBSS. Cell concentration measurements were performed using a Scepter 2.0 Cell Counter (Millipore) and a Countess automated cell counter (Thermo Fisher Scientific). Cell viability was checked with a Countess automated cell counter (Thermo Fisher Scientific). All steps except the DNAse I incubation were performed on ice. For cell capture using the 10× genomics device, the cell concentration was adjusted to 500 cells/µl in HBSS, aiming to capture 5000 cells.

### Single-cell RNA-seq

We followed the manufacturer's protocol (Chromium Single Cell 3′ Reagent Kit, v2 Chemistry) to obtain single cell 3′ libraries for Illumina sequencing. Libraries were sequenced with a NextSeq 500/550 High Output v2 kit (75 cycles) that allows up to 91 cycles of paired-end sequencing: read 1 had a length of 26 bases that included the cell barcode and the UMI; read 2 had a length of 57 bases that contained the cDNA insert; index reads for sample index of eight bases. Cell Ranger Single-Cell Software Suite v1.3 was used to perform sample demultiplexing, barcode processing and single-cell 3′ gene counting using standard default parameters and human build hg19, pig build sus scrofa 11.1 and mouse build mm10. All single-cell datasets that we generated, and the corresponding quality metrics are displayed in Table S6 and were deposited on the Gene Expression Omnibus portal under the series number GSE121600.

### Single-cell quantitative PCR

HAECs were dissociated as described above, then single cells were separated using a C1 Single-cell AutoPrep system (Fluidigm), followed by quantitative PCR on the Biomark system (Fluidigm) using SsoFast evaGreen Supermix (Biorad) and the primers described in Table S7.

### RNA-seq on dissociated and non-dissociated HAECs

Two Transwells from fully differentiated HAECs from two distinct donors were each dissociated as described above. After the final resuspension, cells were centrifuged and resuspended in 800 µl Qiazol (Qiagen). Non-dissociated cells from two Transwells were also lyzed in 800 µl Qiazol. RNAs were extracted with the miRNeasy mini kit (Qiagen) according to the manufacturer's instructions. Two micrograms from each RNA was used in RNA-seq library construction with the Truseq stranded total RNA kit (Illumina). Sequencing was performed with a NextSeq 500/550 High Output v2 kit (75 cycles). Reads were aligned against hg19 human build using STAR aligner. Low expressed genes were filtered out, then paired differential analysis was performed with DESeq2, comparing dissociated versus non-dissociated samples from cultures generated from two different donors. *P*-values were adjusted for multiple testing using the false discovery rate (FDR). Top differentially expressed genes were selected using the following cutoffs: FDR<0.001 and an absolute log2FC>1.5.

### Cytospins

Fully differentiated HAECs were dissociated by incubation with 0.1% protease type XIV from *Streptomyces griseus* (Sigma-Aldrich) in HBSS (Hanks' balanced salts) overnight at 4°C. Cells were gently detached from the Transwells by pipetting and then transferred to a microtube. Cells were then cytocentrifuged at 72 ***g*** for 10 min onto SuperFrost Plus slides using a Shandon Cytospin 4 cytocentrifuge. Cytospin slides were fixed for 10 min in 4% paraformaldehyde at room temperature or with methanol for 10 min at −20°C for further immunostaining.

### Tissue processing for embedding

Nasal turbinates were fixed in paraformaldehyde 4% at 4°C or with methanol at −20°C (for the following antibodies: KRT7, KRT19, DEUP1, centrin 2, HES6) overnight then extensively rinsed with phosphate-buffered saline (PBS). Fixed tissues where then prepared for paraffin embedding or cryo-embedding for cryostat sectioning. For cryoprotection, tissues were soaked in a 15% sucrose solution until saturation of the tissue followed by saturation in a 30% sucrose solution. Tissue was embedded in optimal cutting temperature (OCT) medium (Thermo Fisher Scientific) at room temperature and then submerged in isopentane previously tempered at −80°C. Fully differentiated air-liquid cell cultures were embedded in paraffin using a similar protocol with a shorter time for paraformaldehyde 4% fixation (15 min at room temperature). Each Transwell was cut with a razor blade before embedding. Cutting of frozen tissues was performed with a cryostat Leica CM3050 S. Cutting of paraffin-embedded sections was performed using a rotary microtome MICROM HM 340E (Thermo Fisher Scientific).

### Immunostaining

Samples were permeabilized with 0.5% Triton X-100 in PBS for 10 min. Cells were blocked with 3% BSA in PBS for 30 min. The incubation with primary antibodies was carried out at 4°C overnight. Cells were blocked with 3% BSA in PBS for 30 min. The incubation with primary antibodies was carried out at 4°C overnight. Primary antibodies were as follows: mouse monoclonal anti-KRT4 (1:50, Santa Cruz Biotechnology, sc-52321 for Fig. 4 or 1:250 Proteintech 16572-1-AP for Fig. S11A), rabbit polyclonal anti-KRT5 (1:2000, Biolegend, BLE905501), mouse monoclonal anti-KRT7 (1:100, Dako, M7018), mouse monoclonal anti-KRT8 (1:50, Santa Cruz Biotechnology, sc-58737), mouse monoclonal anti-KRT13 (1:200, Sigma-Aldrich clone KS-1A3), rabbit polyclonal anti-KRT19 (1:250, Proteintech, 10712-1-AP), rabbit polyclonal anti-DEUP1 (1:500, Proteintech, 24579-1-AP), rabbit polyclonal anti-CC10 (SCGB1A1) (1:500, Millipore, 07-623), mouse monoclonal anti-acetylated tubulin (1:500, Sigma-Aldrich clone 6-11B-1), mouse monoclonal anti-MUC5AC (1:250, Abnova clone 45M1), mouse monoclonal anti-SNAI2 (1:50, Santa Cruz Biotechnology, sc-166476), rabbit polyclonal anti-SAP30 (1:200, Proteintech, 27679-AP), goat polyclonal anti-FST (1:200, R&D Systems, AF-669) mouse monoclonal anti-centrin 2 (1/250e, clone 20H5, Sigma-Aldrich, 04-1624) and mouse monoclonal anti-FOXJ1 (1:200, eBiosciences, 14-9965-80).

Secondary antibodies used were: Alexa Fluor 488 goat anti-rabbit (1:500; Thermo Fisher Scientific, A-11008), Alexa Fluor 647 goat anti-mouse (1:500; Thermo Fisher Scientific, A-21235), Alexa Fluor 488 goat anti-mouse IgG1 (1:500, Fisher Scientific, A-21121), Alexa Fluor 594 goat anti-mouse IgG2a (1:500, Fisher Scientific, A-21135), Alexa Fluor 647 goat anti-mouse IgG2b (1:500, Fisher Scientific, A-21242) and Alexa Fluor 488 donkey anti-goat (1:500; Thermo Fisher Scientific, A-11055). Incubation with secondary antibodies was carried out for 1 h at room temperature. Nuclei were stained with 4,6-diamidino-2-phenylindole (DAPI).

When necessary, acetylated tubulin, Muc5AC and KRT5 antibodies were directly coupled to CF 594, 488 and 488 respectively, using the Mix-n-Stain kit (Sigma-Aldrich) according to the manufacturer's instructions. Coupled primary antibodies were applied for 2 h at room temperature after secondary antibodies had been extensively washed and after a 30 min blocking stage in 3% normal rabbit or mouse serum in PBS. MTEC immunostaining was directly performed on Transwell membranes using a similar protocol. For mounting on slides, Transwell membranes were cut with a razor blade and mounted with ProLong Gold medium (Thermo Fisher Scientific). Images were acquired using the Olympus Fv10i or Leica sp5 confocal imaging systems.

### Time course sample analysis

#### Preprocessing

For each sample, cells with levels in the top 5% or bottom 5% of distribution for the following quality metrics: number of expressed features, dropout percentage and library size (total UMI count) were filtered out. Additionally, cells with a percentage of mitochondrial genes >top 5% were also removed. Quality metrics were computed using the scatter package (2.3.0) (McCarthy et al., 2017). Only genes detected (1 UMI) in at least five cells were kept for analysis.

#### Normalization

The scran package (Lun et al., 2016 preprint) was used to calculate cell-based scale factors and normalize cells for differences in count distribution. Each sample was normalized separately twice, first in an unsupervised manner, then after grouping cells of similar gene expression based on our robust clustering results.

#### Clustering robustness

In order to best determine the key steps in the differentiation process, a customized method was implemented to analyze clustering robustness to dataset perturbation. For all possible numbers of clusters (from 2 to 9), multiple subsets of the studied datasets were created (10 subsets with 10% of the cells randomly removed each time) and clustering was performed multiple times on each subset with changing settings of the seed parameter. The result of those clusterings were stored in a (*n* cells)² stability matrix, containing for each pair of cells 1 or 0 depending on whether the cells are clustered together (1) or not (0). This stability matrix was then transformed in a Euclidean distance matrix between cells and then divided into the used k number of clusters k using hierarchical clustering (hclust with ‘average’ method). To identify the optimal number of clusters, a visual inspection of the elbow plot of the average intra-stability (mean stability within each cluster) and the average inter-stability (mean stability between each cluster) was carried out. Cells with a stability metric less than 70% were labeled as ‘unassigned’, owing to the high clustering variability between each round of clustering, then removed from further analysis of the time course data. Cell clustering was performed using SIMLR (package version 1.4.1) (Wang et al., 2017). Heatmaps for the clustering of each dataset are shown in Table S8.

#### Differential analysis

To further analyze the robustness of each step of the differentiation process, we tested the robustness of the cell type marker gene identification through differential gene expression analysis. Differential expression analysis was performed using edgeR (package version 3.22) (Robinson et al., 2010). In a one versus all differential analysis, a pool of 100 cells from one cluster were analyzed against an equal mixture of cells from all other clusters. In a one versus one differential analysis, pools of cells of the same size were compared. Those differential analysis were performed multiple times (10 times) on different pool of cells and the DEG identified were compared between each pool of cells using the rank-rank hypergeometric overlap algorithm (Plaisier et al., 2010). This approach was too stringent and only identified highly expressed marker genes that are less sensitive to dropout events. Thus, the Seurat FindAllMarkers function based on a non-parametric Wilcoxon rank sum test was used to identify cell type marker genes.

#### Time points aggregation

10× datasets generated during the time course were aggregated using MNN correction (Haghverdi et al., 2018) from the scran package.

#### Trajectory inference

Trajectory inference was performed using monocle 2 (package version 2.8) (Qiu et al., 2017). Cell ordering was based on highly variable genes (∼200-500 genes) selected by their expression dispersion. Monocle analysis on the aggregated time points was carried out on raw counts after library size correction (downsampling). Branch building was performed using BEAM analysis from Monocle, and corresponding differential analysis was carried out after a cross comparison of a group of cells along the pseudotime (before branching, after branching and at the branch end) using Seurat 1 versus 1 differential analysis.

#### Cell type projection

To compare cell types identified in distinct samples, cells were projected from one dataset onto the other using scmap R package version 1.1, scmapCluster function (Kiselev et al., 2018).

#### Data visualization

All graphs were generated using R (ggplot2). Heatmaps were obtained using pheatmap (no clustering used, genes ordered by their expression in pseudotime or in cluster, cells ordered by pseudotime or cluster). Heatmaps show smoothed gene expression values: for each gene, normalized gene expression values were first transformed into z-scores, then averaged across 10 neighboring cells in the chosen ordering (pseudotime only or pseudotime in clusters). Single gene representation: for the sake of clarity, only cells with expression levels above the top 50 percentiles for that gene are represented.

### Individual sample analysis

Each sample of our study was reanalyzed with less stringent parameters to identify rare or transitory cell types or gene expression events

#### Preprocessing, normalization and clustering

Individual dataset analysis was performed using Seurat standard analysis pipeline. Briefly, cells were first filtered based on number of expressed features, dropout percentage, library size and mitochondrial gene percentage. Thresholds were selected by visually inspecting violin plots in order to remove the most extreme outliers. Genes expressing fewer than five UMI across all cells were removed from further analysis. Cell-level normalization was performed using the median UMI counts as a scaling factor. Highly variable genes were selected for following analyses based on their expression level and variance. PCA analysis was performed on those genes, the number of PCs to use was chosen upon visual inspection of the PC variance elbowplot (∼10 to 20 PCs depending on the dataset). Clustering was first performed with default parameters and then by increasing the resolution parameter above 0.5 to identify small clusters (but with the knowledgeable risk of splitting big clusters due to high gene expression variability). Differential analysis was again performed using Seurat FindAllMarkers and FindMarkers functions based on non-parametric Wilcoxon rank sum test. Gene Set Enrichment analysis was performed using fgsea R package with the following gene sets reactome.db (R package) and GO cellular component (Broad Institute GSEA MSigDB) genesets. Molecular function enrichment analysis was performed using Ingenuity Pathway Analysis (Qiagen).

#### Cell type annotation

Based on the time course experiment analysis and associated top ∼15 marker genes identified, a score was computed to associate cell types to each cluster. The scoring method is based on Macosko et al. cell cycle phase assignment (Macosko et al., 2015). For each cell it measures the mean expression of the top marker genes for each possible cell type, which results in a matrix c cell types per *n* cells. Then it calculates a z-score of the mean expression for each cell; the top resulting score gives the matching cell type.

#### Velocity

RNA velocity was calculate using latest release of velocyto pipeline (velocyto.org/) using standard parameters: GTF file used for Cell Ranger analysis and the possorted_genome_bam.bam, Cell Ranger output alignment file. From the loom file that contains a count table of spliced and unspliced transcripts, the gene.relative.velocity.estimates function was used on cell type marker genes. The resulting expression pattern of unspliced-spliced phase portraits shows the induction or repression of those marker genes from one cell type to the next. We used velocyto package version 0.5 (La Manno et al., 2018).

### Trajectory inference using Palantir algorithm

Palantir analysis was used as an integrated function of the Scanpy workflow (Wolf et al., 2018). The filtered raw count matrix was loaded into Scanpy, along with the cell type annotation (Scanpy v1.4, Python 3.7); each cell was normalized to the total count over all genes (without log transform) before running Palantir (Setty et al., 2019). The first 14 principal components were used to compute the diffusion map. The corresponding t-SNE embedding was obtained using the first two diffusion components. A start cell was randomly selected among the cycling basal cell cluster to infer trajectories and the associated terminal states. In the process, each cell of the dataset was associated with a probability to differentiate into each of the terminal states identified. Associated with the identified trajectory, Palantir allowed the associated gene trends to be studied using MAGIC (van Dijk et al., 2018) correction of the count matrix.

### Plasscheart et al. dataset

Plasscheart et al.’s data (Plasschaert et al., 2018) were downloaded as processed data along with visualization coordinates and were used without further manipulation. (kleintools.hms.harvard.edu/tools/springViewer_1_6_dev.html?datasets/reference_HBECs/reference_HBECs).

## Supplementary Material

Supplementary information
