# Supplementary material for: Novel dynamics of human mucociliary differentiation revealed by single-cell RNA sequencing of nasal epithelial cultures
Source: Development. 2019 Oct 23;146(20):dev177428. doi: 10.1242/dev.177428 (PMC6826037; doi:10.1242/dev.177428)
Supplement: Supplementary information [file develop-146-177428-s1.pdf]

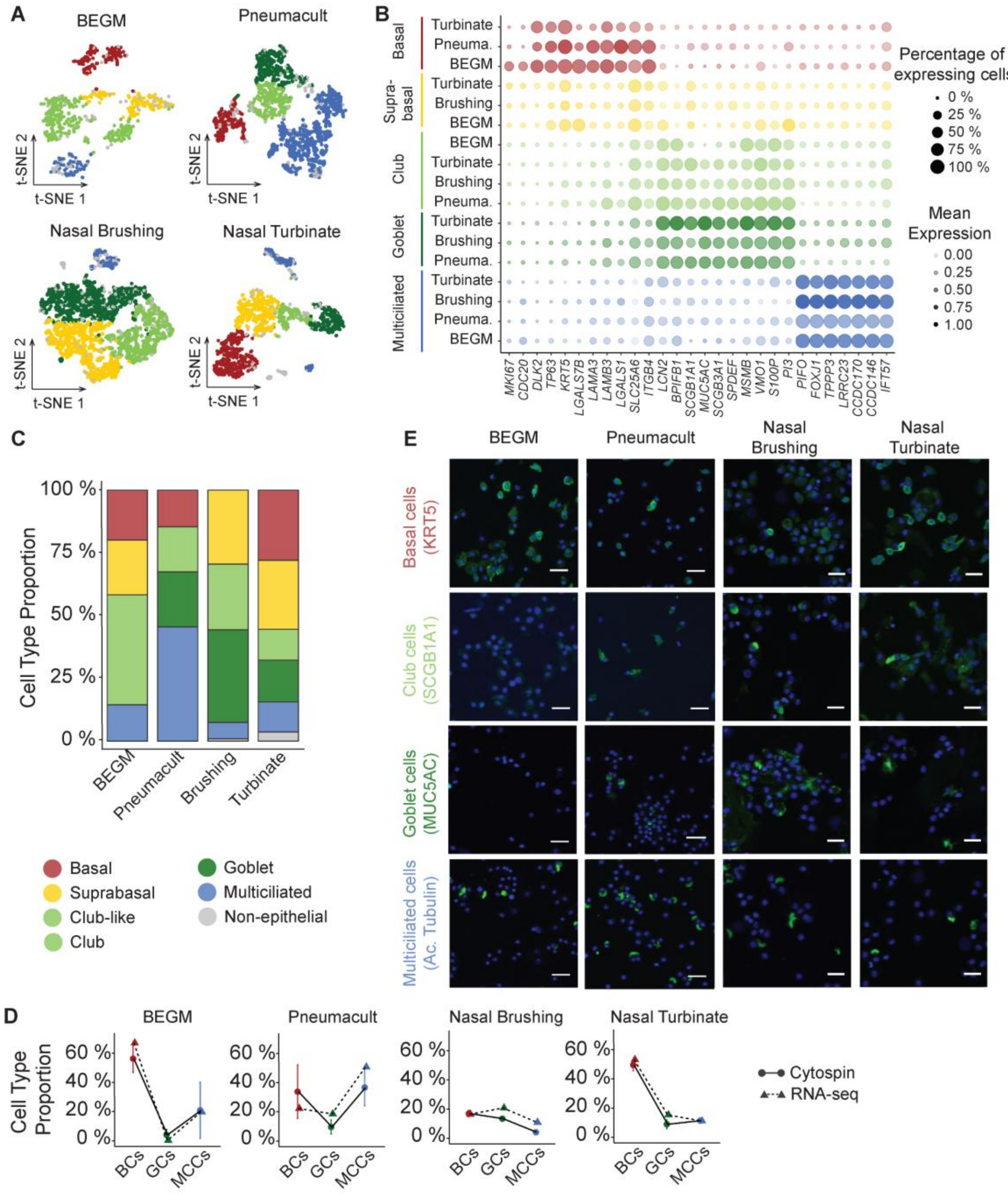

Fig. S1. Cell type composition comparison between homeostatic *in vitro* samples and fresh human tissues

(A) t-SNE representing the distinct cell populations identified in each sample. (B) Dot plot of the main cell population marker genes. Dot size describe the percentage of cells expressing the respective marker genes and the average expression level of that gene based on UMI counts are shown by color intensity. (C) Relative abundance of cell types in each sample. (D) Scatter plot of the percentage of cells expressing the selected marker genes for basal, goblet and multiciliated cells at the transcript (dotted line) and protein level (full line). (E) Immunostaining of the selected marker genes for basal, secretory, goblet and multiciliated in cytospin for each sample. Scale bar: 50 µm.

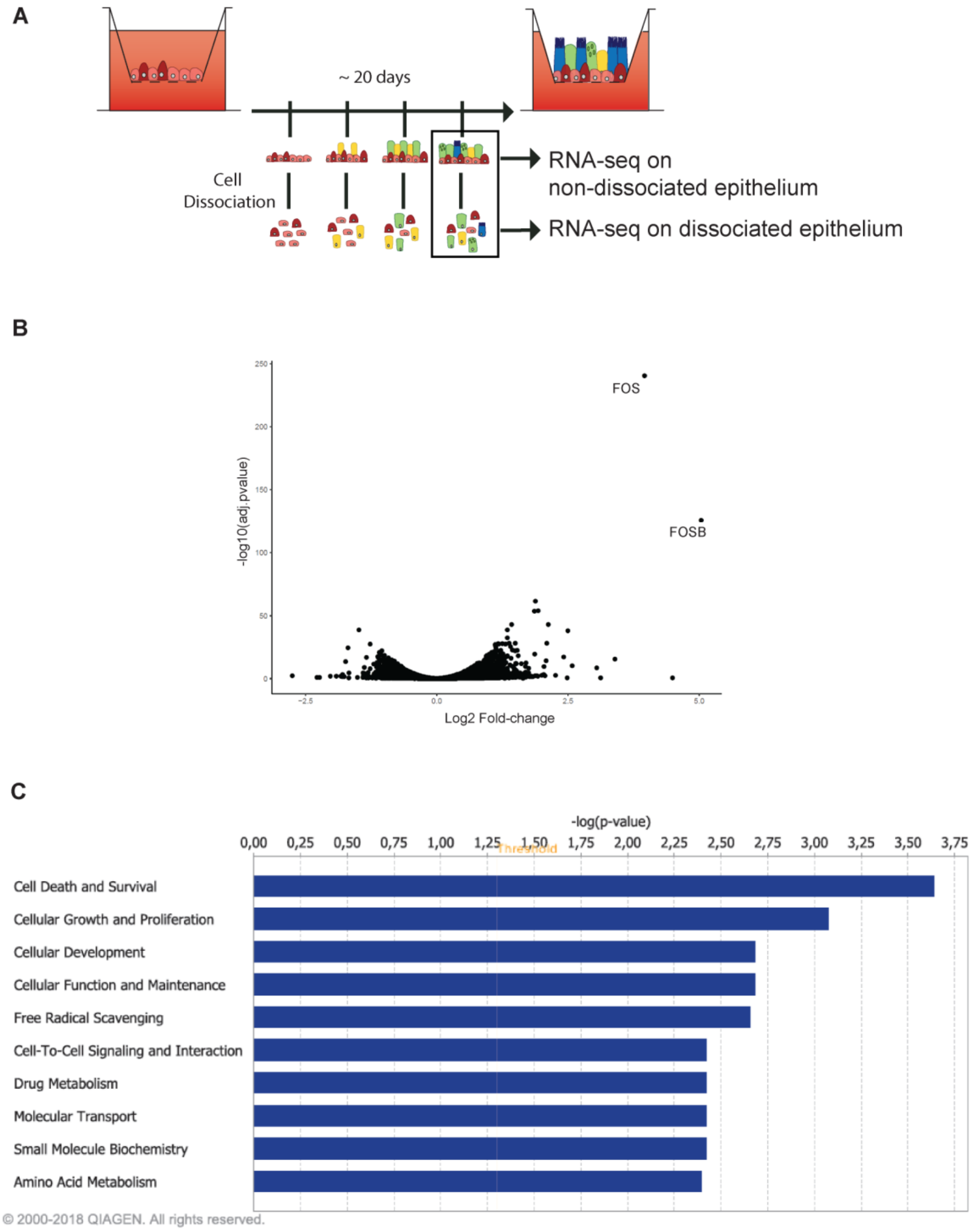

Fig. S2. Effect of cell dissociation on HAEC gene expression

(A) RNA-seq experimental design. Regenerating airway epithelia was dissociated at 28 days after a transition to an air-liquid interface. Dissociated and non-dissociated cultures from 2 donors were subjected to RNA-seq. (B) Volcano plot showing differential gene expression of dissociated vs. non-dissociated cell cultures. (C) Identification, with Ingenuity Pathway Analysis (Qiagen) of the molecular functions most significantly affected by dissociation, based on an analysis of 300 differential expressed genes (FDR<0.01 and abs(log2FC)>1).

Development • Supplementary information

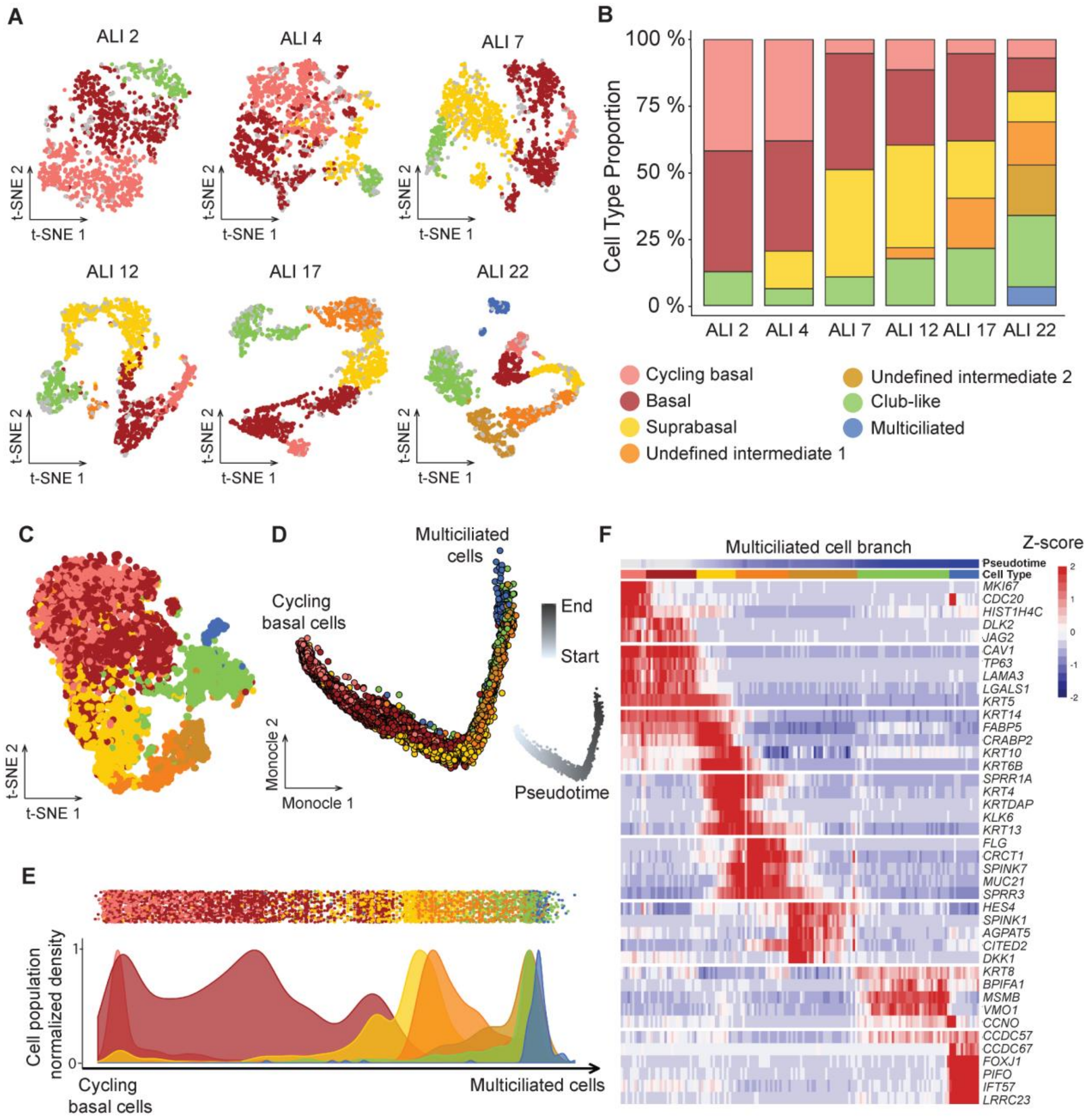

Fig. S3. Characterization of MCC cell lineages during airway epithelium regeneration using single cell RNA-Seq in BEGM medium

(A) t-SNE representing the distinct cell populations identified at each time points. (B) Relative abundance of cell types at each time point. (C) t-SNE plot of the aggregate of all cells from each time point. (D) Representation of the cell lineages inferred by Monocle 2 occurring during the upper airway epithelium regeneration (aggregate of all time points). Pseudotime evolution along the differentiation trajectory shown by white to grey gradient. (E) Distribution of the defined cell types in pseudotime. (F) Heatmap representing the smoothed temporal expression pattern of indicated cell type specific marker genes on MCC trajectory. Cells were ordered by cluster appearance in pseudotime.

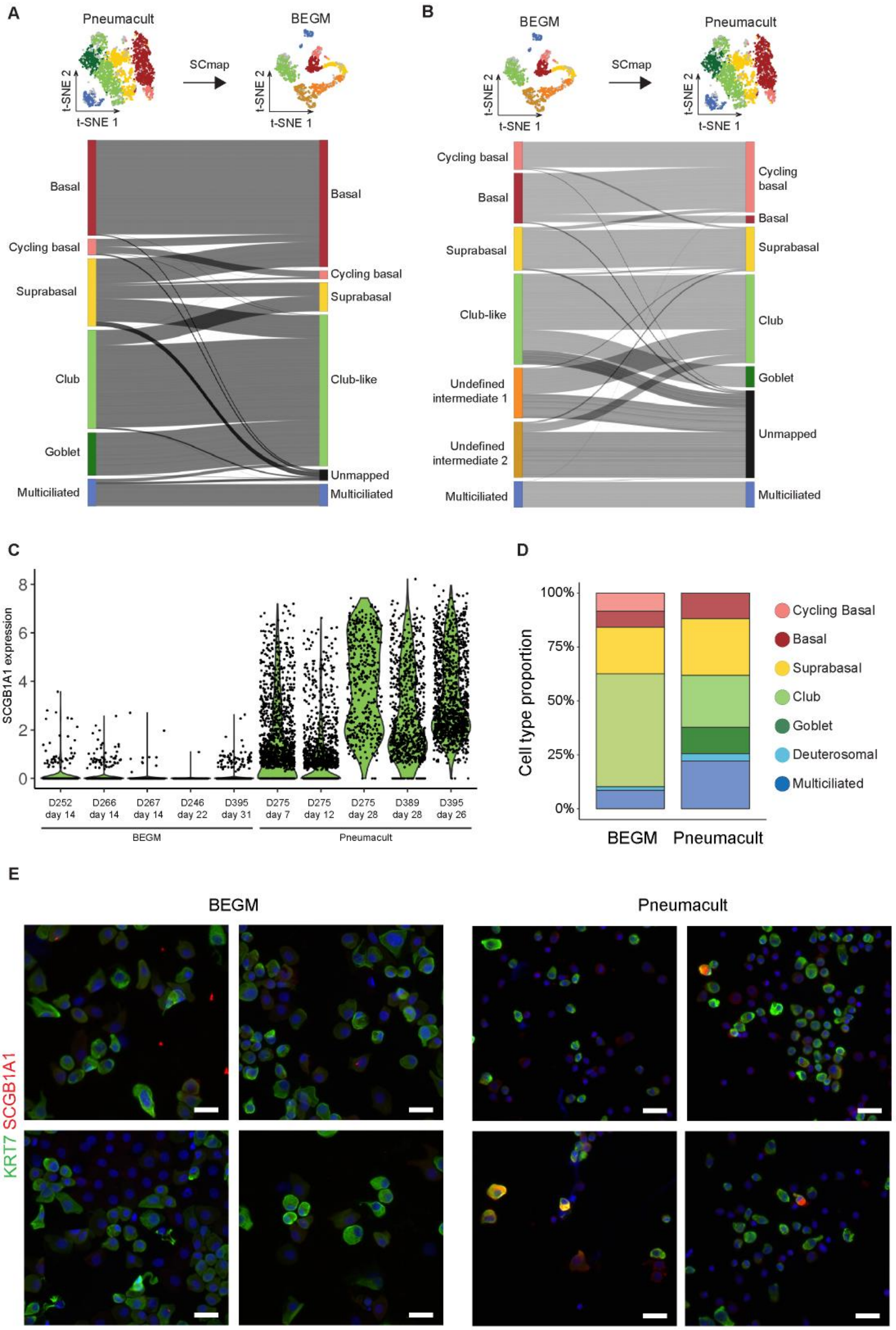

Development • Supplementary information

Fig. S4. Comparison of fully differentiated epithelia cell population between Pneumacult and BEGM media

(A) Sankey Network of the mapping of Pneumacult cells onto BEGM cells. (B) Sankey Network of the mapping of BEGM cells onto Pneumacult cells. (C) *SCGB1A1* expression in single-cell analysis of ALI cultures arising from 5 donors, either differentiated in BEGM medium, or in Pneumacult medium. (D) Cell proportions identified from single-cell RNAseq analysis in cells from the same donor differentiated in BEGM (Day 31) and Pneumacult (Day 26) media. (E) Immonostaining for SCGB1A1 of cells from the same donor differentiated in BEGM (Day 31) and Pneumacult (Day 26). ALI cultures were dissociated and cytospinned for better visualization. KRT7 was also stained as marker of apical/luminal cells.

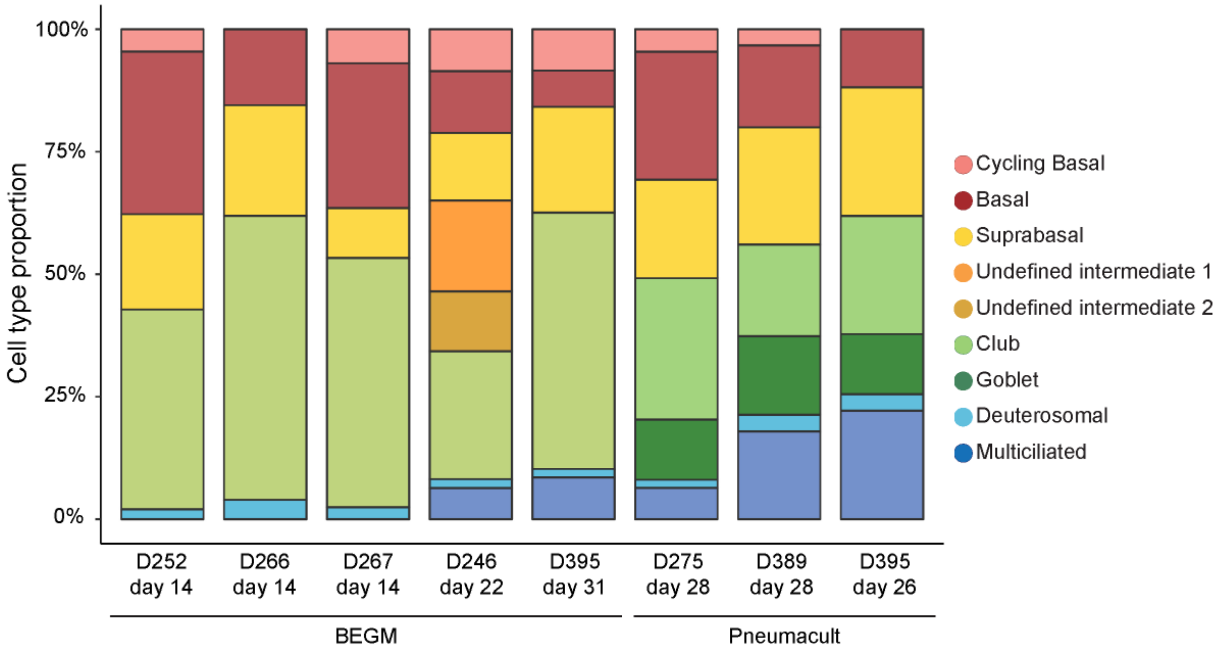

Fig. S5: Cell population distribution from single-cell RNAseq analysis of 8 ALI cultures from 7 independent donors, in BEGM or Pneumacult medium, at several time points. Undefined intermediates have only been identified in one ALI culture.

Development • Supplementary information

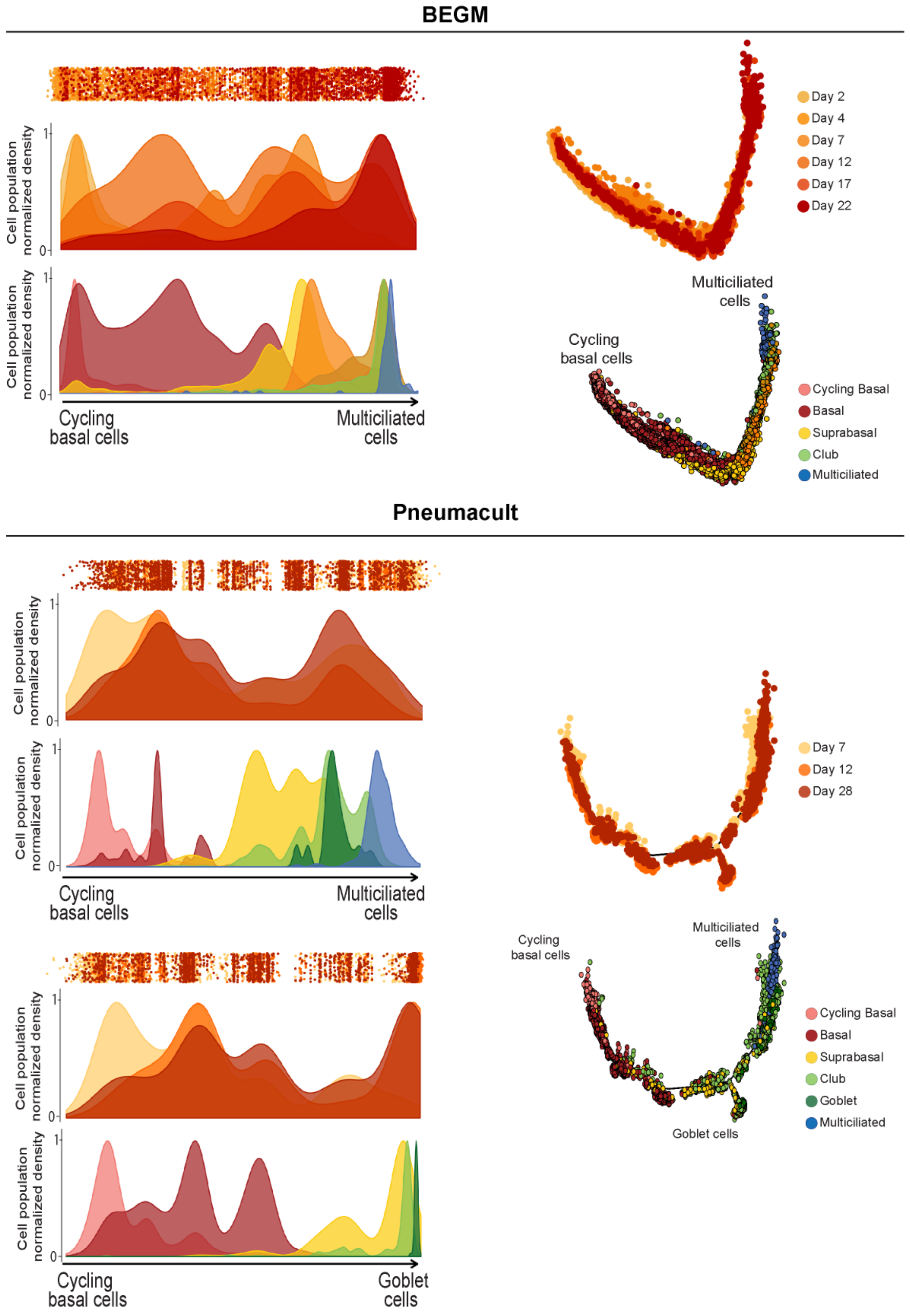

Fig. S6. Trajectory and pseudotime reconstructions color coded by isolation time-point in BEGM and Pneumacult media (aggregate of all time points).

Development • Supplementary information

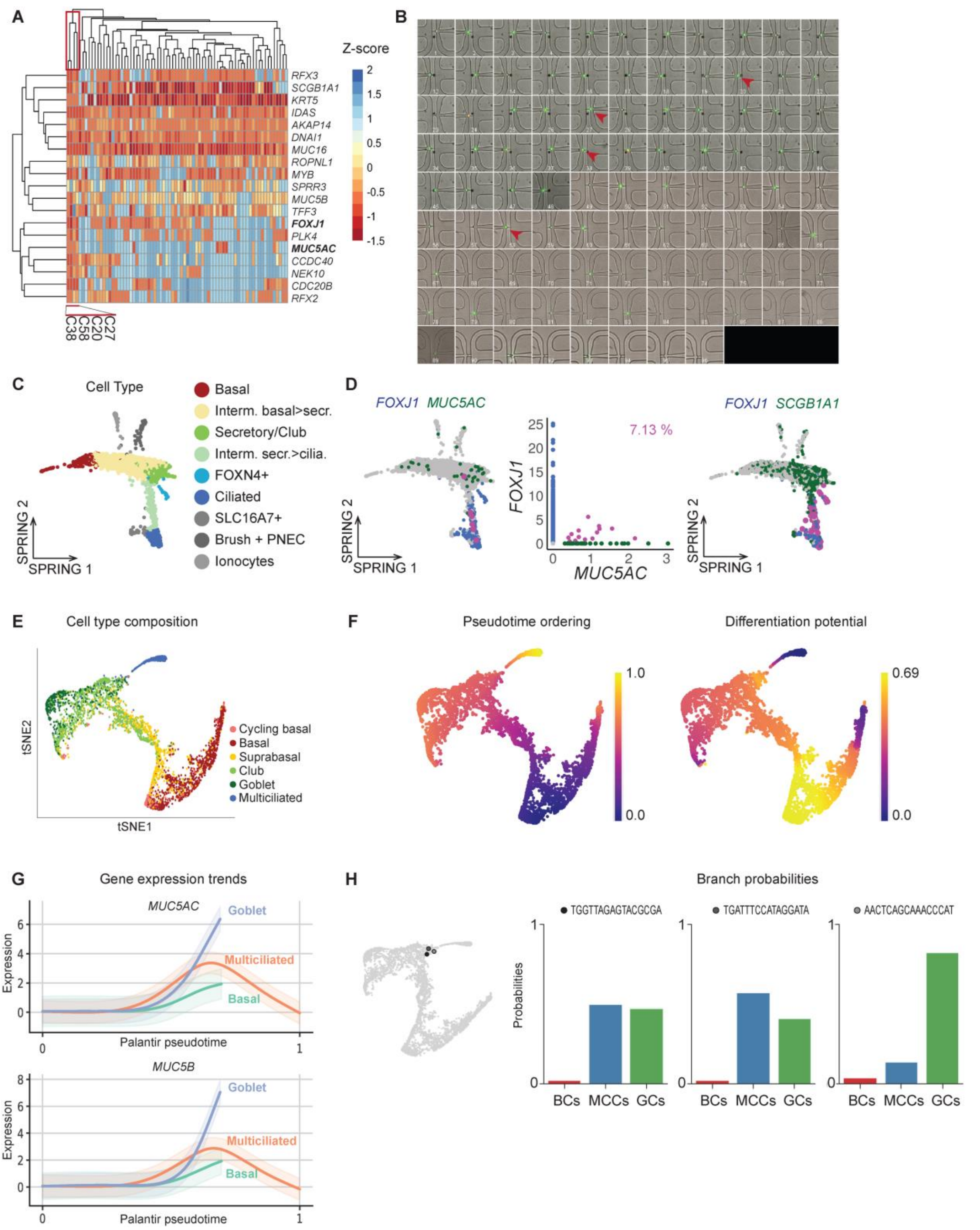

Fig. S7. Validation of Goblet cells markers in multiciliated cells in doublet free single cell transcriptomic datasets

(A) Heatmap colored by Z-score from C1 Biomark experiment in PneumaCult media, co-expression of Goblet and Multiciliated cell markers in single cells (red cluster). (B) C1™ Single-Cell Preamp IFC (10–17 µm) imaging, Red arrowheads show cells expressing both cell type markers (*FOXJ1* and *MUC5AC*),

Development • Supplementary information

one cell per chamber, green cells=living cells, red cells=dead cells. (C) SPRING representations of Plasschaert *et al.* dataset colored by cell type. (D) SPRING representations of Plasschaert *et al.* dataset colored. Left panel colored by *FOXJ1*+ cells (blue), *MUC5AC*+ cells (green), co-expressing *FOXJ1* and *MUC5AC* cells (pink). Center panel displays a scatter plot of normalized expression of *MUC5AC* and *FOXJ1* in cells (dot) colored by *FOXJ1*+ cells (blue), *MUC5AC*+ cells (green), co-expressing FOXJ1 and MUC5AC cells (pink). Right panel colored by *FOXJ1*+ cells (blue), *SCGB1A1*+ cells (green), co-expressing *FOXJ1* and *SCGB1A1* cells (pink). (E) t-SNE representation of the Pneumacult ALI 28 dataset after analysis by Palantir, colored by cell type. (F) t-SNE representation of the Pneumacult ALI 28 dataset after analysis by Palantir, colored by pseudotime (left panel) or differentiation potential (right panel). (G) Gene expression trends for MUC5AC and MUC5B for basal, goblet, and multiciliated cells, computed by Palantir. (H) Branch probabilities for 3 selected goblet cells. Left panel : t-SNE representation of the Pneumacult ALI 28 dataset after analysis by Palantir, showing the 3 selected goblet cells. Right panel: branch probabilities for the 3 selected cells.

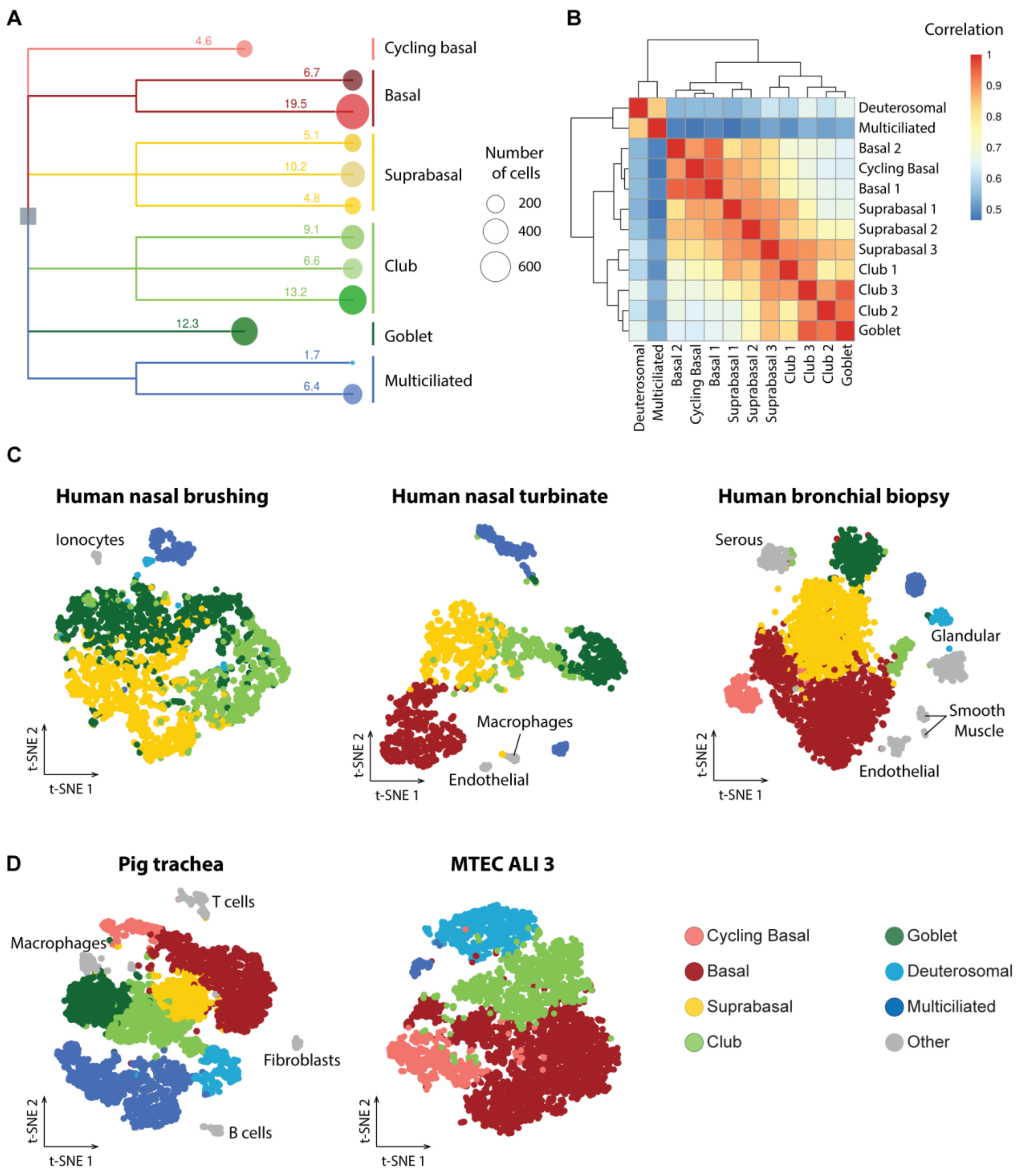

Fig. S8. Identification of subpopulations by high resolution cell clustering

(A) Dendrogram of cell types identified through robust and high-resolution clustering of scRNA-seq data from Pneumacult ALI 28 sample. Numbers indicate percentage of each cluster. (B) Correlation matrix of cell type identified in Pneumacult sample. (C) t-SNE representation of scRNA-seq data from human nasal brushing (left), nasal turbinate (center), bronchial biopsy (right). (D) t-SNE representation of scRNA-seq data from pig trachea (left) and mouse culture (right).

Development • Supplementary information

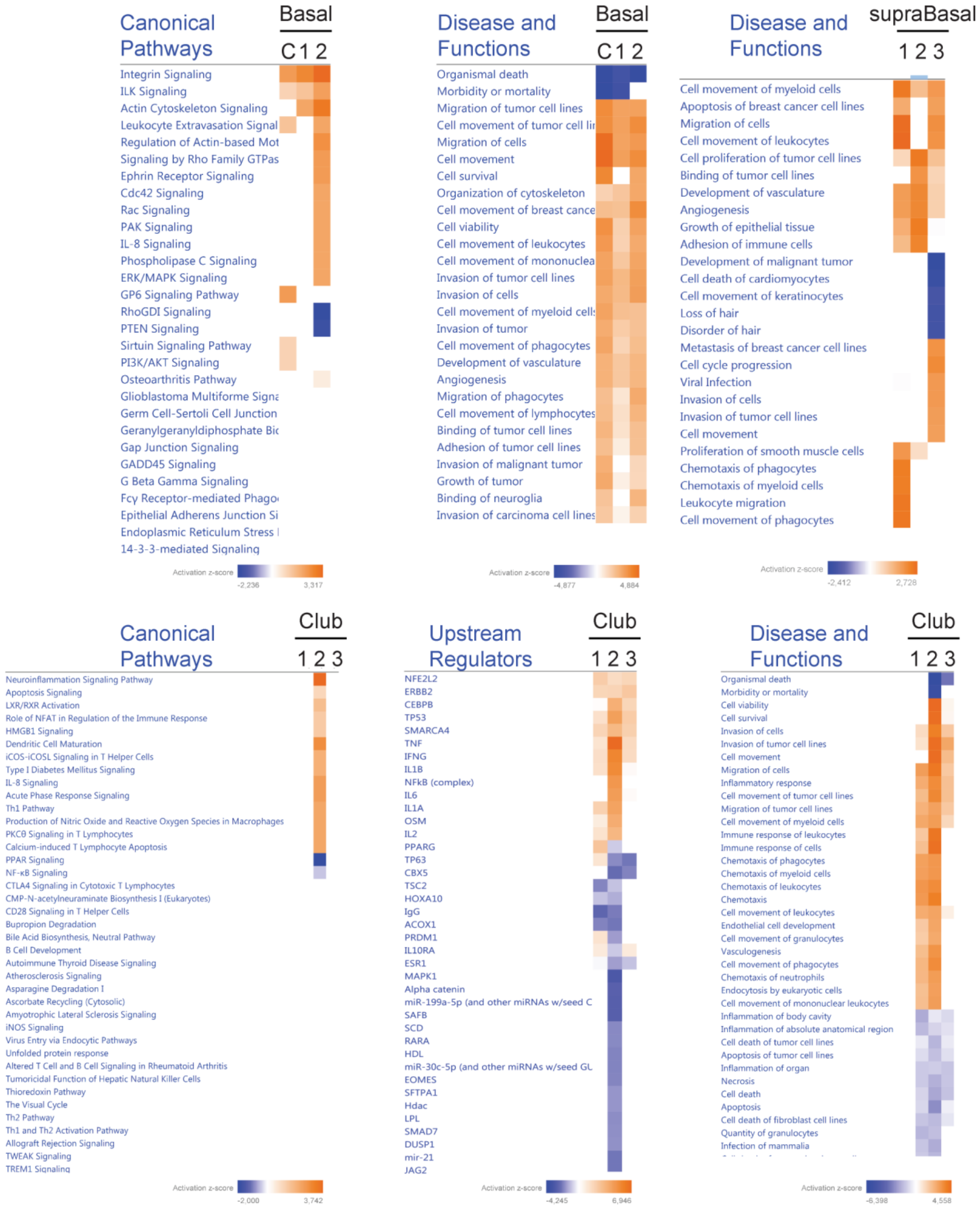

Fig. S9. Pathway enrichment comparison between basal, suprabasal and secretory cell subtypes

Identification, with Ingenuity Pathway Analysis (Qiagen) of the enriched canonical pathways, upstream regulators and disease and functions in each of the basal and secretory subpopulations, based on an analysis of 300 differential expressed genes (FDR<0.01 and log2FC>0.6 for BCs and SCs, and log2FC>0.4 for supraBCs). C: cycling basal cells.

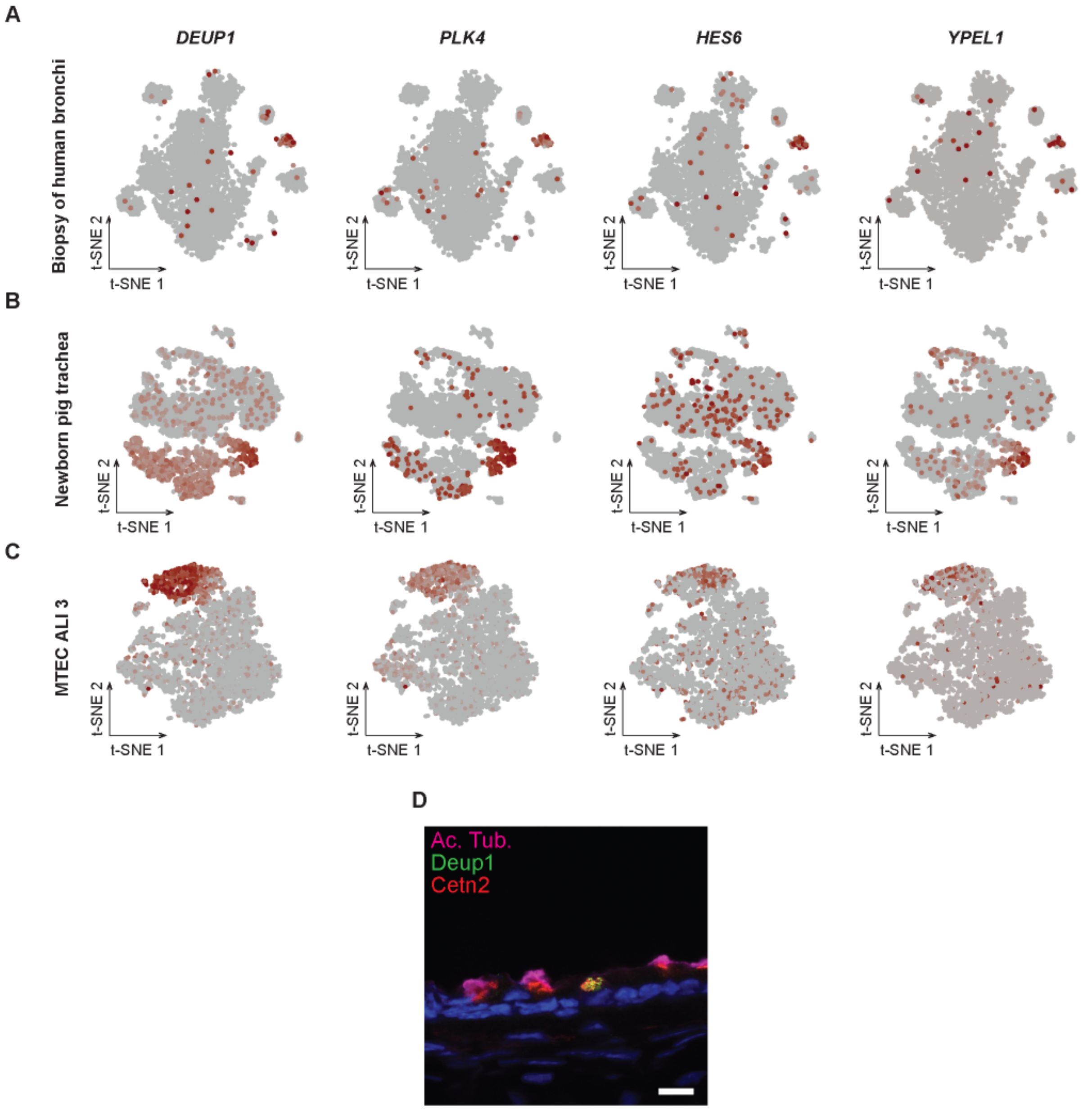

Fig. S10. Robustness of Deuterosomal cells marker genes

(A-C) t-SNE representation of Deuterosomal cell population marker genes expression (lowly to highly expressed, grey to red) in (A) Biopsy of human trachea, (B) Newborn pig trachea, (C) MTECs ALI 3. (D) Immunohistochemistry on a section of adult mouse trachea showing acetylated tubulin, Deup1 and Centrin 2 expression. Scale bar : 10 µm.

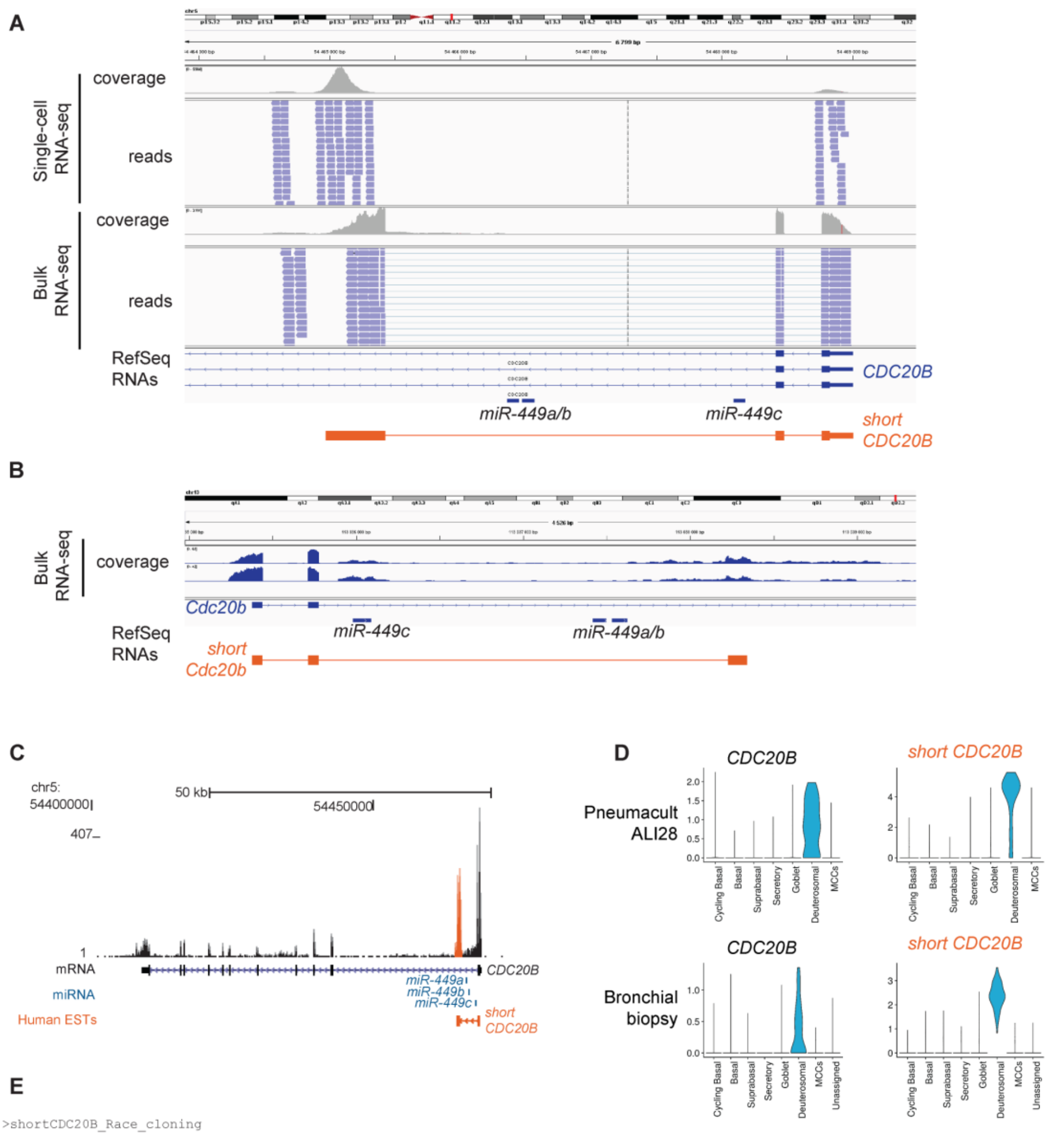

E

```
>shortCDC20B_Race_cloning
AGCGGCGGTGGGAGGAGTGGGGGACCTGGAGGGAGGTGGTGAAATAGAATGGTCCTGGGG-
GACGGGACTCCAGGGGTTTGACGATTAGGCGTCAAGACTGACTTAGACCTGGGAAGAAAACCAGAGGCAGTCGAGCAGAGAGGCCAAACGCCAGGCAGGGCGAAGTCAGCCGGAGATGGAGTGGAAACTGGA
GCGCACCGCGCCTCGGAGGGTCCGCACGGAAGAGGAGATGCTGTGGGAAAGTATCATGCGTGTGCTCTCCAAAGACTTGAAGCAGAAGAGAAGTCAAGATTCCGCCAACGTTCATGAAATATTTGGAAGACA
GCATGAGATCTAACAACTTGTGAAGTGGGAAGAACTGACCATACAATATGACCTTAAATATATTCTCATTACCTTGGAGGCATTTTGACCCACGAAGTAGCATAGATATGTGCTGGCTTCCAGACATGCAGT
ATTCTTGTGCGGACATTTGCAGCCAGTCAGTGGAACTGACTTTGGTGGTGCCATCAGTTAGTTAAGCCATAGATGAGATATAAGAAATATTTCGCATTCTCTACTGTACTGGATTATAAGTTATTGGTAAAA
TAATTTTTATGCTTTCCTTATTTAAACATATTTCTAGAACATGGGTTAAATAGATATCTATAATTTGATTCTTACGCATTTTACTACTTTCAAATCACATCCTCTTTTTGATGTTGCCCAGTGTTATTCACT
TTCAGGAGATTTATGTTTAAGTATATCTTCCATGTAAATATTATTTTCATCTTGTCTTTTTTTAAGGTTAAATTTCAACATGTTATTTTCTATAAAGAATCACTGACCTAGTGATAAAAATAAAACCTACTA
AATTTAGTAAAAAAAAAAAAAAAAAAAAAAAAAAAAAA
```

Fig. S11. Identification of short CDC20B, a novel isoform of a deuterosomal cell population marker gene.

(A) Integrated Genome Viewer (hg19) view of the *CDC20B* gene with coverage and read alignment from bulk RNA-seq of well-differentiated HAECs and single-cell RNAseq of ALI 14 differentiated HAECs from the dataset GSE103518. (B) Genome Viewer (mm10) view of the *Cdc20b* gene with coverage from bulk RNA-seq of ALI 7 differentiated MTECs from the dataset GSE75715. (C) UCSC Genome browser (hg19) view of the CDC20B gene with coverage from read alignment from bulk RNA-seq of ALI28 differentiated HAECs. The alternative 3rd exon is shown in orange. (D) Violin plots for CDC20B and short CDC20B abundance in the Pneumacult ALI28 and the bronchial biopsies samples. (E) Alternative human short CDC20B sequence identified with 5’ race cloning.

Development • Supplementary information

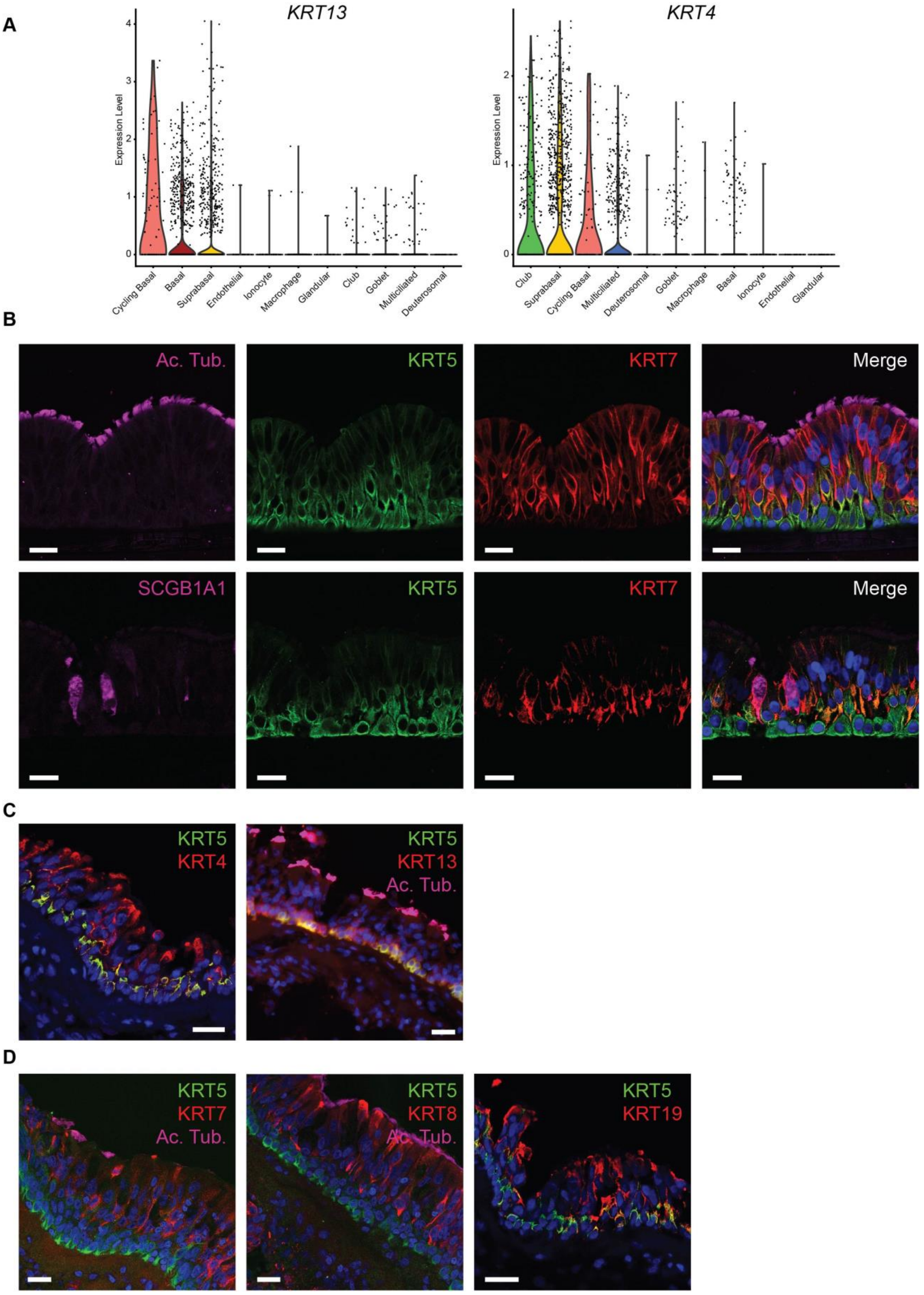
A
KRT13
Expression Level
Cycling Basal
Basal
Suprabasal
Endothelial
Ionocyte
Macrophage
Glandular
Club
Goblet
Multiciliated
Deuterosomal
KRT4
Club
Suprabasal
Cycling Basal
Multiciliated
Deuterosomal
Goblet
Macrophage
Basal
Ionocyte
Endothelial
Glandular
B
Ac. Tub.
KRT5
KRT7
Merge
SCGB1A1
KRT5
KRT7
Merge
C
KRT5
KRT4
KRT5
KRT13
Ac. Tub.
D
KRT5
KRT7
Ac. Tub.
KRT5
KRT8
Ac. Tub.
KRT5
KRT19

Fig. S12. Keratin expression in nasal airway epithelium

(A) Violin plots for KRT13 and KRT4 expression in epithelial cells freshly isolated from a nasal turbinate. (B) Immunohistochemistry for KRT5, KRT7 and Acetylated Tubulin or SCGB1A1 on sections of Pneumacult-fully differentiated *in vitro* epithelium, as in Fig. 4F, with split channels. (C) Immunohistochemistry for KRT5, KRT4 and KRT13 in sections of nasal turbinates. (D) Immunohistochemistry for KRT5, KRT7, KRT8 and KRT19 in sections of nasal turbinates. Cilia are labeled with acetylated tubulin when noted. Scale bars: 20 µm.

Development • Supplementary information

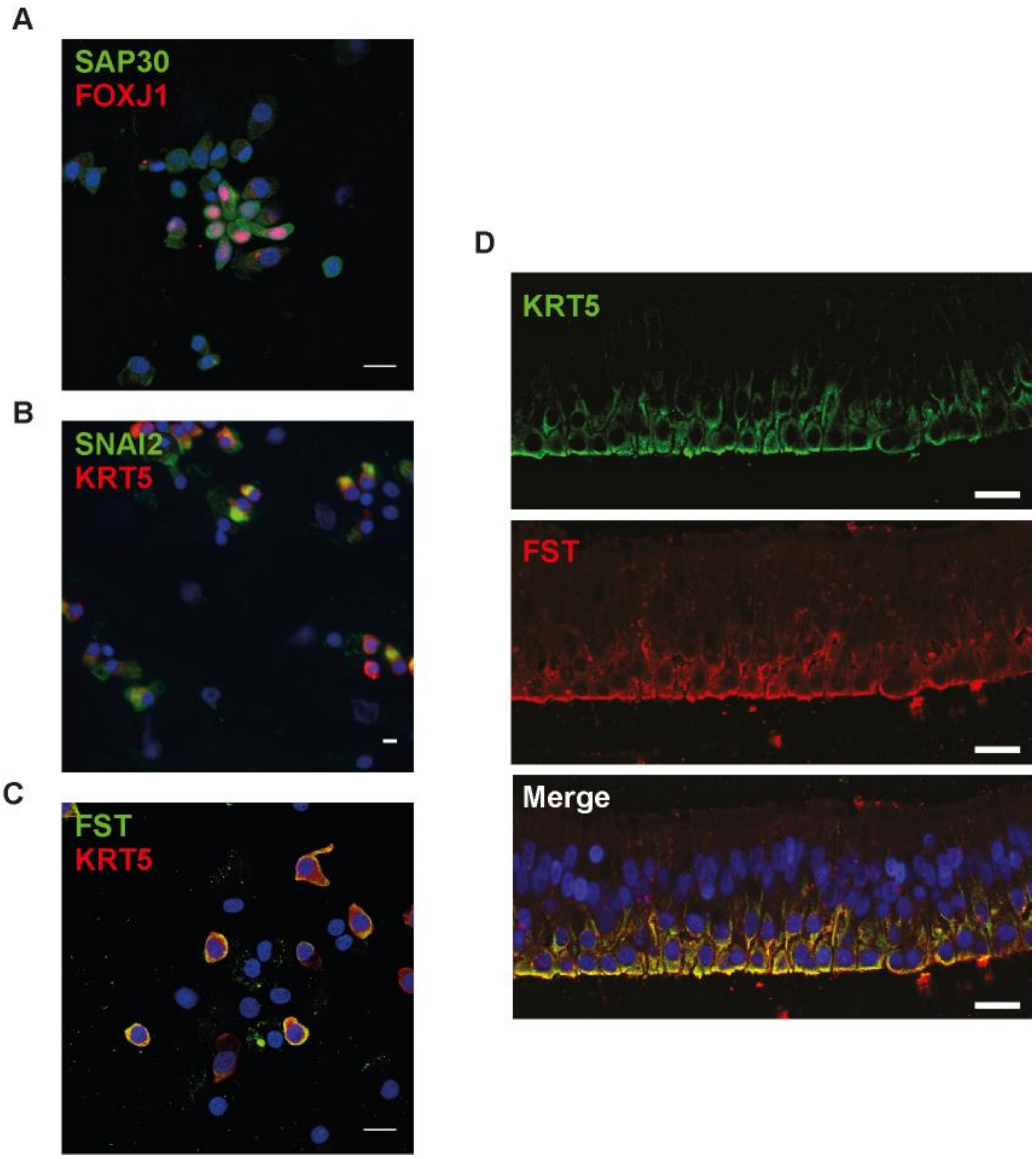

Fig. S13. Validation of expression of signaling pathways components

(A) Immunocytochemistry for SAP30 and FOXJ1 on dissociated Pneumacult-fully differentiated cells. (B) Immunocytochemistry for SNAI2 and KRT5 on dissociated epithelial cells from a nasal brushing. (C) Immunocytochemistry for FST and KRT5 on dissociated Pneumacult-fully differentiated cells. (D) Immunocytochemistry for FST and KRT5 on sections of Pneumacult-fully differentiated cells. Scale bars: 20 µm.

Development • Supplementary information

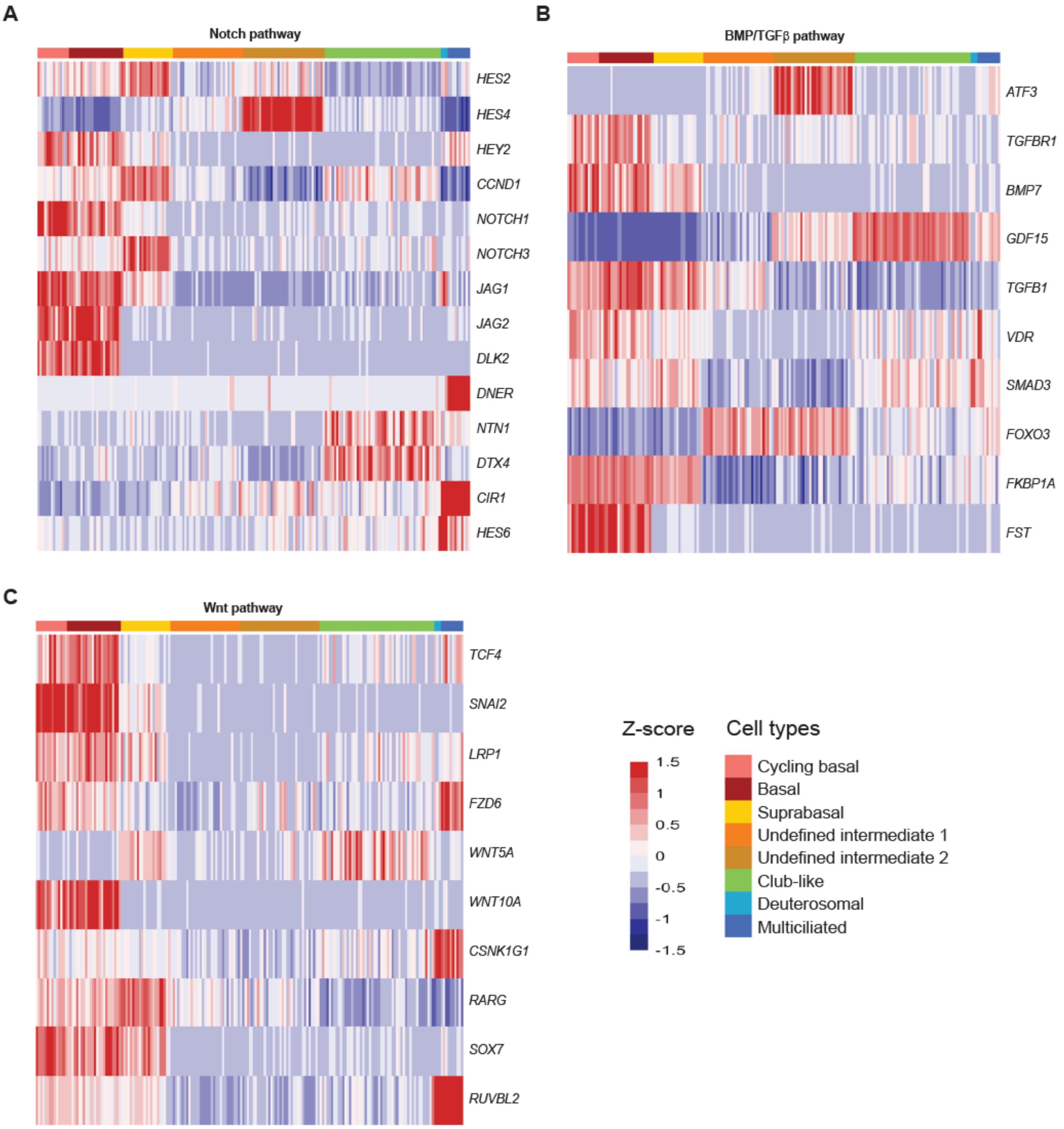

Fig. S14. Expression of signaling pathways components of an ALI culture in BEGM medium

(A) Heatmap of the genes related to the NOTCH pathway. (B) Heatmap of the genes related to the BMP/TGFβ pathway. (C) Heatmap of the genes related to the Wnt pathway. For all heatmaps, cells were ordered by cluster, obtained from the dataset of BEGM-differentiated cells (ALI 22).

Development • Supplementary information

UCSC Cell browser links for data visualization:
http://caire.ipmc.cnrs.fr/cellbrowser/Differentiation/Pneumacult/
http://caire.ipmc.cnrs.fr/cellbrowser/Differentiation/BEGM/

Table S1. Robust cell type markers in the Pneumacult ALI28 sample

Click here to Download Table S1

Table S2. Robust cell type markers in the BEGM ALI22 sample

Click here to Download Table S2

Table S3. Differential Gene expression of club/goblet after branching vs. before branching and differential gene expression of End vs. Beginning of GC branch (2 tabs)

Click here to Download Table S3

Table S4. Detailed cell type markers in the Pneumacult ALI28 sample

Click here to Download Table S4

Table S5. Differential Gene expression of Deuterosomal vs. all other clusters, or of deuterosomal vs.

Click here to Download Table S5

Table S6. Summary of all the single-cell RNA-seq datasets of the study and comparison with quality metrics from other published airway single-cell datasets

Click here to Download Table S6

Development • Supplementary information

Table S7: Primers used in the Biomark qRT-PCR

Gene	Sequence of forward primer	Sequence of reverse primer
AKAP14	AGATTGTGGAAGAGGAGCGAAACCC	TACCATGCTGGAGTGGTCGGC
CCDC40	CATCCCACGGAGTCTTAGGC	TGGATCCTGTCAATCTGCCC
CDC20B	CGGCTGAGAAATATGCTTGG	ATAAACACGCCCCAGTCTTG
DNAI1	AACGACGGCTGTCCCTAAAG	AGCCTACAAAACGCTCCCTC
FOXJ1	TGGATCACGGACAACTTCTG	GAGGCACTTTGATGAAGCAC
IDAS	TTTCAGAGACACGGTGGATG	TGGTGATATGTCGCAAGGAG
KRT5	AGGAATGCAGACTCAGTGGAG	CAGAGGAAACACTGCTTGTGAC
MUC16	GTCAAGCCAGGCAGCACAAGG	GGGATGTGCCTGCTGGACTGC
MUC5AC	CCAAATACGCCAACAAGACC	ATTCCATGGGTGTCAGCTTG
MUC5B	TGCAACCGTCCCGGCTTCG	GTTGTGTTGCACACGCACACCG
MYB	ATGATGAAGACCCTGAGAAGGAAA	AACAGGTGCACTGTCTCCATGA
NEK10	GCAAGAAATCACCATCAGGGAC	GGCTGGAAGCTGTTGTTTGC
PLK4	TCCAACACAGGCACCAATC	GAGATGTCTGTTCCAGAAGCTG
RFX2	GCGACCACATCCTCTACCAG	AGTTACGGATGGCCTGTGTC
RFX3	AGCCAACATCATCAACAGTTTTT	TGCAGTGACTTGATATCCTCAAA
ROPN1L	CTCGCATCCCCTTCAAGACG	TCTTCCTGGCGTCTATATTTTCC
SCGB1A1	AGAGACGGGCCAGAGCATCCC	GGCAGATCTCTGCAGAAGCGGAGC
SPRR3	TCTGCACAGCAGGTCCAGCATC	AGGCTGGCTGGGTTGTTTCACC
TFF3	GGAGTACGTGGGCCTGTCTGC	AGCCCCGGTTGTTGCACTCC

Table S8: Heatmaps for clustering of each dataset

Click here to Download Table S8
